# Growth and acetate metabolism of *Staphylococcus aureus* in defined medium

**DOI:** 10.1128/aem.01554-25

**Published:** 2025-10-16

**Authors:** Fareha Razvi, Taylor L. Burke, Dhananjay Shinde, Jongsam Ahn, Vinai C. Thomas, Marat Sadykov, Paul D. Fey

**Affiliations:** 1 Department of Pathology, Microbiology, and Immunology, University of Nebraska Medical Center 12284, Omaha, Nebraska, USA; Washington University in St. Louis, St. Louis, Missouri, USA

**Keywords:** acetate, metabolism, *Staphylococcus aureus*

## Abstract

*Staphylococcus aureus* is a major human pathogen that has emerged as a model pathogen to study growth and metabolism. Many investigators have designed defined media (complete defined medium; CDM) to investigate staphylococcal genetic and metabolic processes. Previous *S. aureus* growth studies have found that acetate is not consumed following glucose catabolism in certain CDM recipes, nor are arginine and proline consumed, suggesting a defect in post-exponential growth. The current studies found that acetate catabolism is dependent upon the coordinated action of acetyl-CoA synthetase (Acs), the AcuA acetylase, and AcuC deacetylase. Furthermore, it was determined that acetate catabolism in *S. aureus* in CDM is linked to a critical ratio between the concentration of amino acids and glucose. These studies will help inform *S. aureus* metabolic experiments to ensure complete oxidation of carbon sources in the medium and therefore interrogate metabolism of the post-exponential phase.

**IMPORTANCE:**

Growth characteristics are critical to the study of S*taphylococcus aureus* metabolism and genetics. These studies have defined a critical concentration of amino acids and glucose that is required to ensure the complete oxidation of acetate and amino acids found in the medium. The complete defined medium (CDM) described in these studies will be used to inform future studies to ensure phenotypic and genotypic characteristics that are observed during post-exponential growth can be assessed.

## INTRODUCTION

Many studies assessing the genetics and physiology of *Staphylococcus aureus* have used complex media, such as tryptic soy broth (TSB) as a growth medium (1–10). Indeed, critical aspects of *S. aureus* carbon metabolism have been evaluated in TSB, which contains 14 mM glucose and an ill-defined mixture of peptides and free amino acids derived from soybean and casein. For example, during aerobic growth in TSB, glucose is rapidly consumed and acetate is generated by the phosphate acetyltransferase (Pta; SAUSA300_0570) and acetate kinase (AckA; SAUSA300_1657) pathway (6, 11, 12). Consistent with typical observations of overflow metabolism (13, 14), glucose does not undergo complete oxidation in the tricarboxylic acid (TCA) cycle. Instead, excess carbon is redirected toward acetate production, which is then secreted into the medium. Once glucose is consumed, the extracellular acetate is subsequently transported and converted into acetyl-CoA via acetyl-CoA synthetase (AcsA; SAUSA300_1679). Citrate synthase (GltA; SAUSA300_1641) utilizes acetyl-CoA and oxaloacetate as substrates to generate citrate, thus driving the TCA cycle, respiration, and post-exponential growth. Importantly, catabolism of proline and arginine, amino acids that are used as substrates to synthesize glutamate, a critical carbon and oxaloacetate source when glucose is exhausted, is only observed during post-exponential growth due to transcriptional regulation via carbon catabolite repression and CcpA (15). Pertinent to the premise of studies outlined in this manuscript, global regulators that govern carbon catabolism, such as CcpA and CodY, are also linked to multiple biological processes, including virulence factor production (16, 17). Therefore, due to changes in global transcriptomic responses linked to differing growth conditions, a firm understanding of the growth medium and aeration used (18) is critical to experimental design, regardless of the biological property studied.

As studies assessing staphylococcal metabolism have matured, many investigators have developed defined media, typically containing 18 amino acids plus a primary carbon source, such as glucose, to further control their experiments (15, 19, 20). In contrast to other model organisms, such as *Escherichia coli* and *Bacillus subtilis, S. aureus* is phenotypically auxotrophic for certain amino acids, making studies requiring minimal medium difficult, especially those required to define preferred carbon and nitrogen sources. Interestingly, although *S. aureus* has the genetic capability to synthesize all 20 amino acids, certain amino acid pathways are repressed during growth in complex and defined media, and valine and arginine are required for growth irrespective of the growth medium used (15, 21, 22). However, the use of a medium containing a carbon and nitrogen source plus valine and arginine does not support the growth of *S. aureus* (P. D. Fey, unpublished observations). Therefore, since the amino acid requirements for *S. aureus* growth are not completely understood, our laboratory has typically used a complete defined medium (CDM) containing 18 amino acids, excluding glutamine and asparagine (15, 23). During these growth studies in CDM lacking glucose, we found that the secreted acetate via catabolism of the pyruvate generating amino acids was subsequently consumed, similar to that observed in TSB. However, we found that acetate was not consumed if 14 mM glucose (same concentration as found in TSB) was added to CDM (CDMG), nor was post-exponential growth observed or catabolism of proline or arginine. Since many metabolic studies require the use of a defined medium containing glucose, we sought to investigate acetate metabolism during growth in defined medium when glucose and two non-phosphoenolpyruvate:sugar phosphotransferase system (PTS) carbon sources, pyruvate and gluconate, were used as the primary carbon source. We found that a critical concentration of both glucose and amino acids is required to facilitate the complete oxidation of acetate and amino acids (proline and arginine) that fuel glutamate and thus oxaloacetate. These studies will inform and help design future global metabolomic, transcriptomic, and proteomic studies to ensure assessment of post-exponential growth and complete oxidation of amino acids in the medium.

## RESULTS

### Growth of *S. aureus* JE2 in complete defined medium with and without glucose

**Fig 1:**
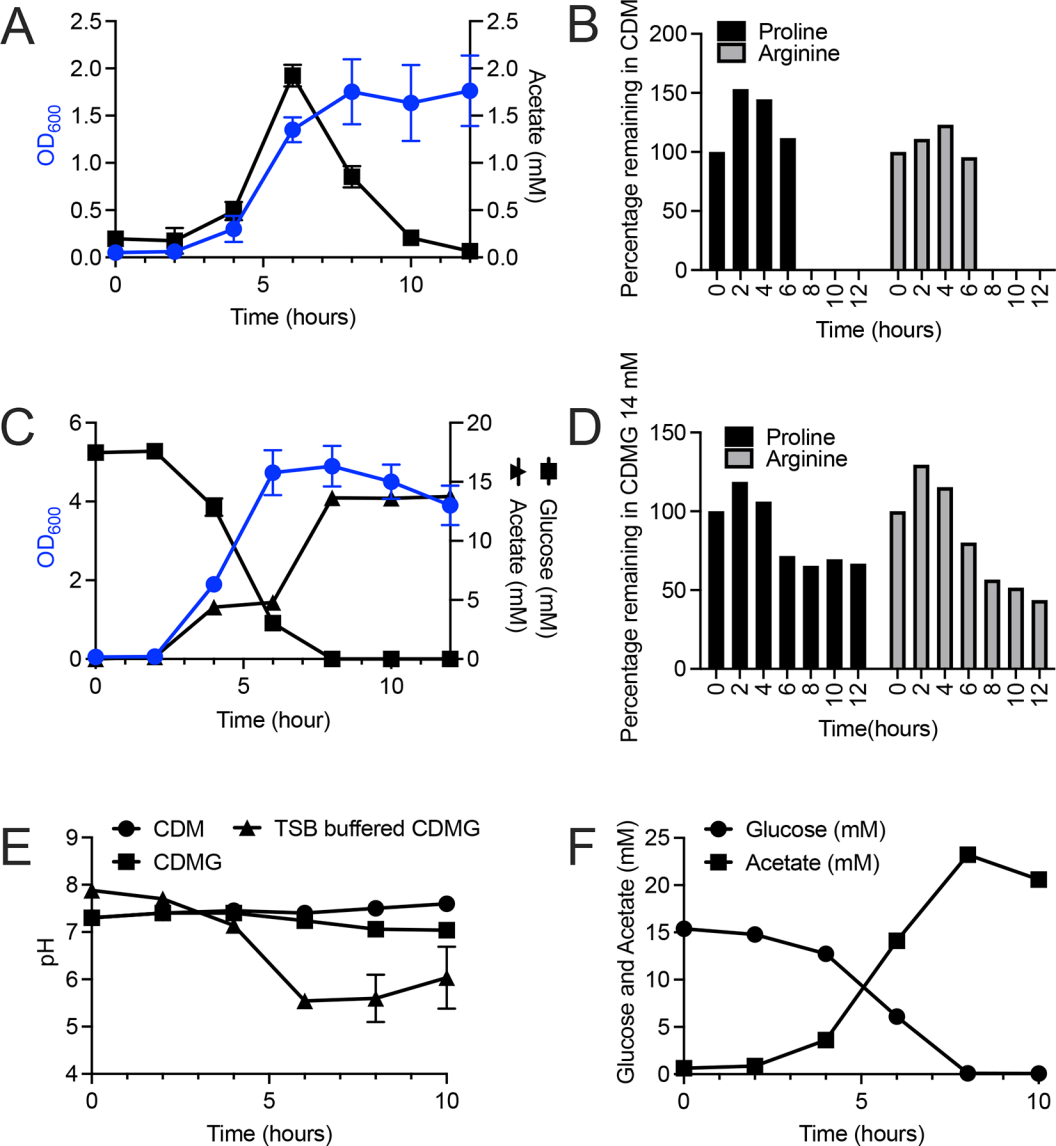
Growth and metabolite analysis of *S. aureus* JE2. (**A**) Aerobic growth (blue) of JE2 in CDM and concentration of acetate in the culture medium during growth (black). (**B**) Percentage of proline and arginine remaining in the CDM following growth at 0, 2, 4, 6, 8, 10, and 12 h. (**C**) Aerobic growth (blue) of JE2 in CDM containing 14 mM glucose (CDMG) and concentration of acetate (black squares) and glucose (black triangles) in the culture medium during growth. (**D**) Percentage of proline and arginine remaining in CDMG following growth at 0, 2, 4, 6, 8, 10, and 12 h. (**E**) Change in pH during aerobic growth of JE2 in CDM, CDMG, or CDMG buffered with 2.5 g/L K_2_HPO_4_ (TSB-buffered CDMG). (**F**) Catabolism of acetate during growth in TSB-buffered CDMG (black) is not observed following glucose catabolism (blue).

We have previously performed studies assessing amino acid catabolism in *S. aureus* using a CDM that contained 18 amino acids but lacked glutamine, asparagine, and glucose (15). When *S. aureus* JE2 was grown aerobically in CDM, acetate was secreted via catabolism of the pyruvate, generating amino acids, presumably threonine, serine, and alanine. In the post-exponential phase, the secreted acetate was subsequently oxidized by the TCA cycle, thus generating reducing power. Furthermore, analysis of the culture supernatant showed rapid catabolism of eight amino acids (serine, glycine, threonine, alanine, arginine, proline, glutamate, and aspartate) during aerobic growth in CDM by 8 h of growth. When 14 mM glucose was added to the chemically defined medium (CDMG), JE2 secreted acetate concomitantly with the catabolism of glucose during the exponential phase. However, depletion of glucose from CDMG was not followed by consumption of acetate or amino acids that fuel glutamate synthesis (proline and arginine). To provide a baseline for our current studies, JE2 was grown aerobically in CDM and CDMG (Table S1), and supernatant was collected for metabolite and amino acid analysis (Fig. 1). Growth in CDM reached a maximum OD_600_ growth yield of 2.0 and secreted ~2.0 mM acetate by 6 h of growth (Fig. 1A). The extracellular acetate was completely consumed by 12 h of growth during post-exponential growth (Fig. 1A). Amino acid analysis of the spent medium showed that proline and arginine, both amino acids that serve to fuel glutamate synthesis, were catabolized by 8 h of growth (Fig. 1B) in addition to serine, threonine, glycine, alanine, glutamate, and aspartate (Fig. S1A and B). The remaining amino acids were slowly catabolized or not used as carbon sources, but most likely were transported for proteinogenic purposes (Fig. S1C through E).

In contrast, growth in CDMG resulted in a maximum OD_600_ growth yield of ~5.0 and produced approximately 15 mM extracellular acetate (Fig. 1C). However, upon depletion of glucose (~8 h), as opposed to growth in CDM and TSB (6), extracellular acetate was not consumed and utilized to drive the TCA cycle. Furthermore, in contrast to growth in CDM and TSB, *S. aureus* JE2 grown in CDMG did not fully catabolize proline or arginine, following exponential growth (Fig. 1D). Similar to growth in CDM, the amino acids serine, threonine, glycine, alanine, glutamate, and aspartate were catabolized by 4–6 h of growth (Fig. S2A and B). The remaining amino acids, cysteine, phenylalanine, tyrosine, isoleucine, leucine, valine, methionine, tryptophan, and histidine were either not catabolized or slowly catabolized (Fig. S2C through E). However, in contrast to growth in CDM, growth in CDMG resulted in catabolism of lysine by 6 h of growth (Fig. S1E and S2E).

It is well documented that as *S. aureus* catabolizes glucose in tryptic soy broth, acetate is secreted into the culture medium, and the pH typically shifts to ~5.5 (4, 12). However, upon acetate consumption, the pH increases to ~7.2 presumably due to the consumption of acetate and NH_3_^+^ production via amino acid catabolism (4, 12). These observations led us to hypothesize that the enhanced buffering capacity of CDMG, in contrast to TSB, represses acetate catabolism in CDMG. To address if a reduction in pH is required to induce acetate catabolism, the buffering capacity found in TSB (2.5 g/L K_2_HPO_4_) was used to replace the strong buffering capacity found in CDM (10.0 g/L Na_2_HPO_4_ and 3.0 g/L KH_2_PO_4_). Using this medium (TSB-buffered CDMG), accumulation of acetate in media resulted in a pH shift from 7.8 to 5.4 by 6 h (Fig. 1E). However, this reduction in pH did not result in subsequent acetate catabolism following depletion of 14 mM glucose from TSB-buffered CDMG (Fig. 1F).

### Acetate catabolism in CDM is *acsA* and *acuC* dependent

A study by Burkhardt et al. using *S. aureus* HG001 found that acetate catabolism in *S. aureus* is mediated by acetyl-CoA synthetase (AcsA; SAUSA300_1679) and its activity is modulated by reversible acetylation at a conserved lysine residue (24–26). Additionally, it was determined that acetyltransferase activity via AcuA (SAUSA300_1680) renders AcsA inactive, whereas activation of AcsA is mediated by deacetylation performed by the NAD^+^-dependent (class III) sirtuin protein deacetylase enzyme CobB (SAUSA300_2157). However, *S. aureus* encodes multiple deacetylases, including AcuC (SAUSA300_1681; found just downstream of *acuA*) and SirTM (SAUSA300_0327), both of which are interrogated in our study.

**Fig 2:**
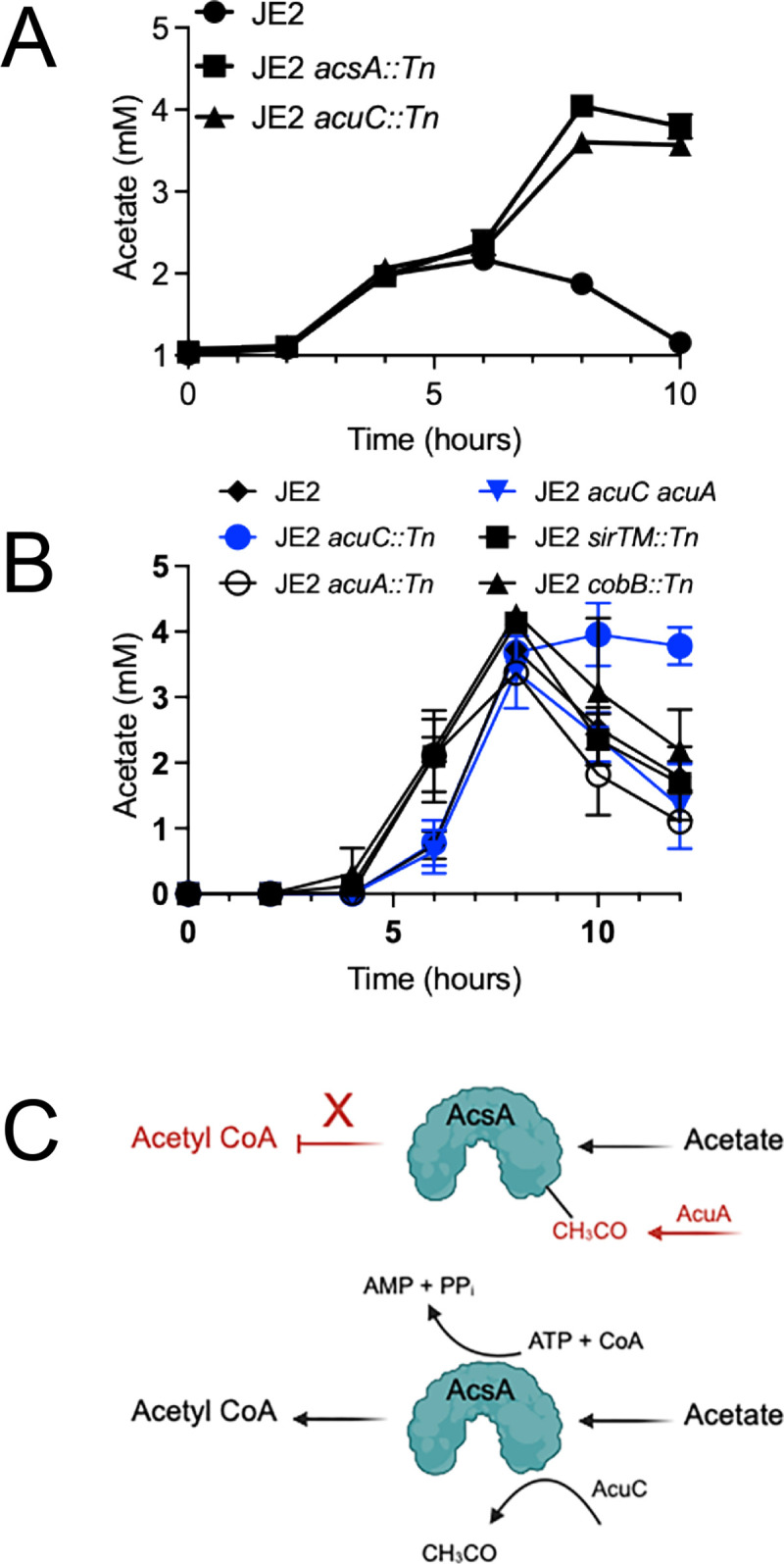
Acetate catabolism of *S. aureus* JE2 grown in CDM is dependent upon the coordinated activity of AcsA, AcuA, and AcuC. *S. aureus* JE2, JE2 *acsA::Tn,* and JE2 *acuC::Tn* were grown in CDM, and acetate concentration was assessed in the growth medium from 0 to 10 h (**A**). JE2, JE2 *acuC::Tn,* JE2 *acuA::Tn,* JE2 *acuC::Tn acuA::tmp,* JE2 *sirTM::Tn,* and JE2 *cobB::Tn* were grown in CDM, and acetate concentration was assessed in the growth medium from 0 to 10 h (**B**). Model of AcsA function following acetylation via AcuA and deacetylation by AcuC (**C**).

Previously described acetylation/deacetylation experiments by Burkhardt et al. were performed using well-developed *Salmonella* tools as a heterologous host (24, 26). Therefore, to attain further experimental evidence of AcsA-mediated acetate catabolism during growth of *S. aureus* in CDM, JE2 *acsA::Tn* was grown in CDM, and acetate consumption was ascertained in comparison to JE2. Indeed, these growth experiments documented that JE2 *acsA::Tn* was unable to catabolize extracellular acetate in comparison to JE2 (Fig. 2A). Furthermore, JE2 *acuA::Tn* completely catabolized acetate during growth in CDM and thus phenocopied JE2 (Fig. 2B). This was not surprising, as inactivation of *acuA,* encoding the acetylase that inactivates AcsA, should not repress acetate catabolism. However, we predicted that acetate catabolism would be dependent upon a specific deacetylase that activates AcsA activity. In contrast to studies performed in *Salmonella* as a heterologous host (24)*,* impaired acetate catabolism was observed when JE2 *acuC::Tn* was grown in CDM (Fig. 2A) but not *cobB::Tn* (Fig. 2B). In addition, acetate catabolism was not affected during growth of *sirTM::Tn* (Fig. 2B). Next, since we surmised that acetate catabolism was defective in JE2 *acuC::Tn* due to an inability to deacetylate, and thus activate AcsA, we predicted that introducing an *acuA* mutation in JE2 *acuC::Tn* should restore acetate catabolism. As shown in Fig. 2B, acetate catabolism was indeed restored in JE2 *acuC::Tn acuA::Tn* as compared to JE2 *acuC::Tn*. These results suggest that during growth of *S. aureus* in CDM, AcsA facilitates catabolism of acetate, whereas AcuC functions as a deacetylase, thus activating AcsA. Hence, acetate catabolism during growth in CDM is due to the coordinated activity of the acetyl-CoA synthetase AcsA, the acetyltransferase AcuA, and the deacetylase AcuC (Fig. 2C).

### Acetate catabolism is independent of CcpA and CodY regulation in CDMG

**Fig 3:**
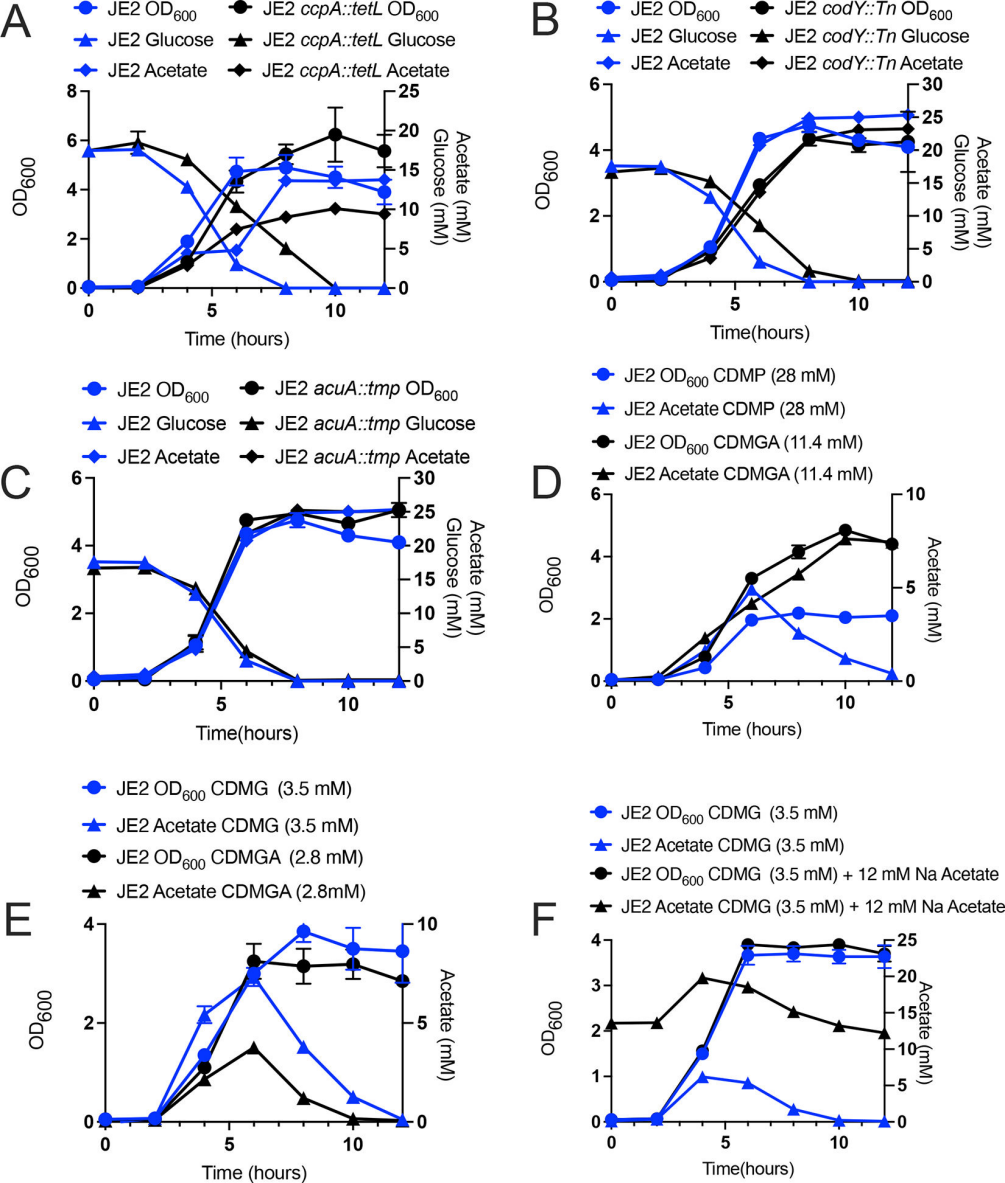
Growth of *S. aureus* JE2 in CDM containing different carbon sources. (**A to C**) Growth, acetate production, and glucose consumption of *S. aureus* JE2 *ccpA::tetL* (**A**), JE2 *codY::Tn* (**B**), and JE2 *acuA::Tn* (**C**) in comparison to JE2. (**D**) OD_600_ and extracellular acetate concentration of JE2 following growth in CDM containing 28 mM pyruvate (CDMP) or CDM containing 11.4 mM gluconic acid (CDMGA). (**E**) OD_600_ and extracellular acetate concentration of JE2 following growth in CDM containing 3.5 mM glucose (CDMG) or CDM containing 2.8 mM gluconic acid (CDMGA). (**F**) OD_600_ and extracellular acetate concentration of JE2 following growth in CDM containing 3.5 mM glucose with and without 12 mM sodium acetate.

These results led us to speculate that the impaired catabolism observed in CDMG could be due to an inactive AcsA or downregulation of *acsA* transcription. Indeed, growth of *B. subtilis* in media containing glucose represses *acsA* and *acuABC* in a CcpA-dependent manner (27). Therefore, in order to determine if defective acetate catabolism in CDMG was due to CcpA repression of *acsA*, JE2 *ccpA::tetL* (28) was grown in CDM containing 14 mM glucose (CDMG) to the exponential phase (OD_600_ of 0.9–1.0), and samples were collected for RNA extraction. In agreement with previous studies in *B. subtilis* (27), the transcript of *acsA,* in addition to *acuA* and *acuC*, was increased >10-fold in JE2 *ccpA::tetL* in comparison to JE2 (Fig. S3). However, although JE2 *ccpA::tetL* produced less acetate than JE2 (Fig. 1C), it failed to utilize acetate upon glucose depletion, suggesting that the increased *acsA* transcription as noted in JE2 *ccpA::tetL* does not function to enhance acetate catabolism during growth in CDMG (Fig. 3A). Furthermore, in *B. subtilis, ackA* encoding acetate kinase is positively regulated by another global transcriptional regulator CodY (29). To determine if acetate catabolism is regulated by CodY, JE2 *codY::Tn* (SAUSA300_1148) was grown in CDMG, and acetate consumption was assessed. However, inactivation of *codY* did not result in acetate consumption as compared to WT JE2 (Fig. 3B). We next assessed whether post-translational modification of AcsA via AcuA may function to regulate acetate catabolism during growth in CDMG. We predicted that by inactivating AcuA, which acetylates and thereby impairs AcsA activity, we might rescue acetate catabolism during growth in CDMG. Hence, we grew JE2 *acuA::*Tn in CDMG and assayed growth, as well as extracellular acetate, and we found that inactivation of *acuA* did not result in a growth defect nor restore active acetate catabolism (Fig. 3C).

To further explore acetate catabolism in CDM, we grew JE2 in CDM containing the non-PTS carbon sources pyruvate (CDMP; 28.0 mM) and gluconic acid (CDMGA; 11.4 mM). These studies found that acetate was only consumed during growth in CDMP (Fig. 3D). The growth yield in CDM containing 28 mM pyruvate was lower than in CDM with either 11.4 mM gluconic acid (Fig. 3D) or 14 mM glucose (Fig. 1C), likely due to the reduced contribution of glycolysis to ATP production. Since a reduced growth yield facilitated acetate catabolism in both CDM and CDMP, we hypothesized that reducing the concentration of glucose or gluconic acid would facilitate acetate catabolism. Therefore, JE2 was grown in CDM containing either 3.5 mM glucose or 2.8 mM gluconic acid (Fig. 3E); using both these growth conditions, we observed that acetate was indeed catabolized, although less acetate was produced during growth in CDMGA (2.8 mM) as compared to CDMG (3.5 mM) (Fig. 3E). Furthermore, since growth in CDMG (14 mM) resulted in high concentrations of extracellular acetate, we wanted to determine if these growth conditions inhibited acetate catabolism. Therefore, JE2 was grown in CDMG (3.5 mM glucose) containing 12.0 mM sodium acetate. No difference in growth rate or yield was noted when JE2 was grown in CDMG (3.5 mM) with or without 12 mM sodium acetate (Fig. 3F). Importantly, the increased concentration of exogenous acetate (12.0 mM) did not inhibit acetate excretion, which was a byproduct of glucose catabolism and its subsequent utilization in post-exponential phase by JE2 (Fig. 3F).

**Fig 4:**
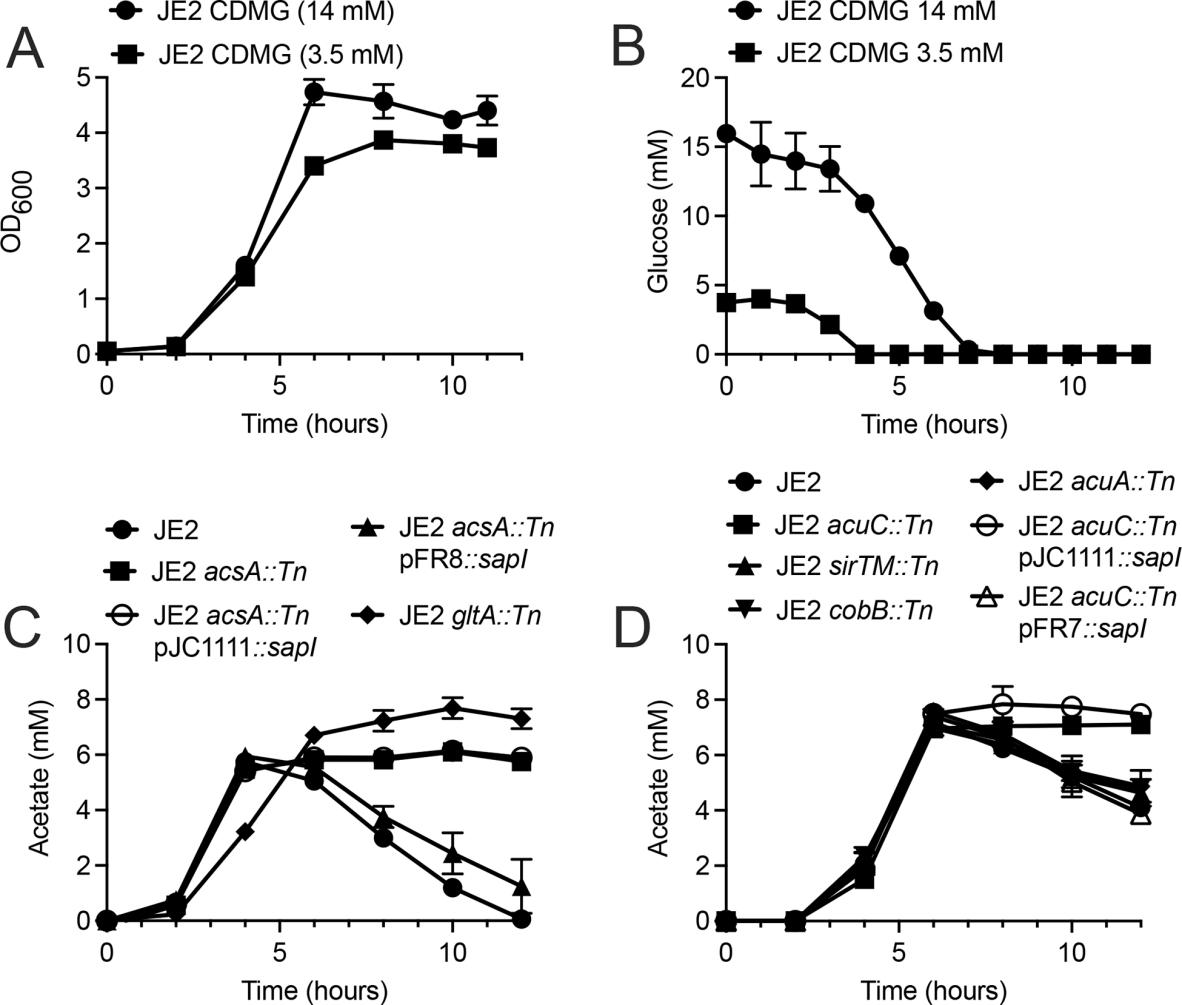
Acetate production during growth in CDMG (3.5 mM glucose). (**A**) Growth of JE2 in CDMG with either 14 or 3.5 mM glucose. (**B**) Glucose concentration in the extracellular medium following growth in either CDMG with either 14 or 3.5 mM glucose. (**C**) Concentration of acetate in the growth medium following growth of JE2, JE2 *acsA::Tn*, JE2 *gltA::Tn,* JE2 *acsA::Tn* pJC1111::SaPI1*,* and JE2 *acsA::Tn* pFR8::SaPI1 in CDMG (3.5 mM glucose). Note the acetate consumption when the *acsA* mutant was complemented with *acsA* integrated into the SaPI1 integration site in comparison to the empty vector (pJC1111). (**D**) Concentration of acetate in the growth medium following growth of JE2, JE2 *acuC::Tn,* JE2 *acuA::Tn,* JE2 *sirTM::Tn,* and JE2 *cobB::Tn,* JE2 *acuC::Tn* pJC1111::SaPI1*,* and JE2 *acuC::Tn* pFR7*::*SaPI1 in CDMG (3.5 mM glucose). Note the acetate consumption when the *acuC* mutant was complemented with *acuC* integrated into the SaPI1 integration site in comparison to the empty vector (pJC1111).

 Further growth experiments were performed to more fully interrogate the growth of JE2 in CDMG containing 14 or 3.5 mM glucose. As expected, an enhanced growth rate and higher growth yield were observed when JE2 was grown in CDM containing 14 mM glucose as compared to 3.5 mM (Fig. 4A). Glucose was depleted by 4 and 7 h during growth in CDM containing 3.5 and 14 mM glucose, respectively (Fig. 4B). Furthermore, we found that acetate catabolism during growth in CDM containing 3.5 mM glucose is dependent upon *acsA,* similar to JE2 *gltA::Tn* (citrate synthase, SAUSA300_1641) which cannot utilize acetate during post-exponential growth due to a defect in the TCA cycle (12) (Fig. 4C). Furthermore, as anticipated, JE2 *acuC::Tn* exhibited impaired acetate catabolism, suggesting that deacetylation and subsequent inactivation of AcsA is dependent upon AcuC during growth in CDM containing 3.5 mM glucose (Fig. 4D). Lastly, restoration of acetate catabolism was observed when JE2 *acsA::Tn* and *acuC::Tn* were complemented via insertion of *acsA* (Fig. 4C) or *acuC* (Fig. 4D) into the SaPI insertion site using either the native promoter (*acsA*) or a non-cognate *sarA* constitutive promoter (*acuC*).

### Proline and arginine are catabolized during growth in CDM containing 3.5 mM glucose

**Fig 5:**
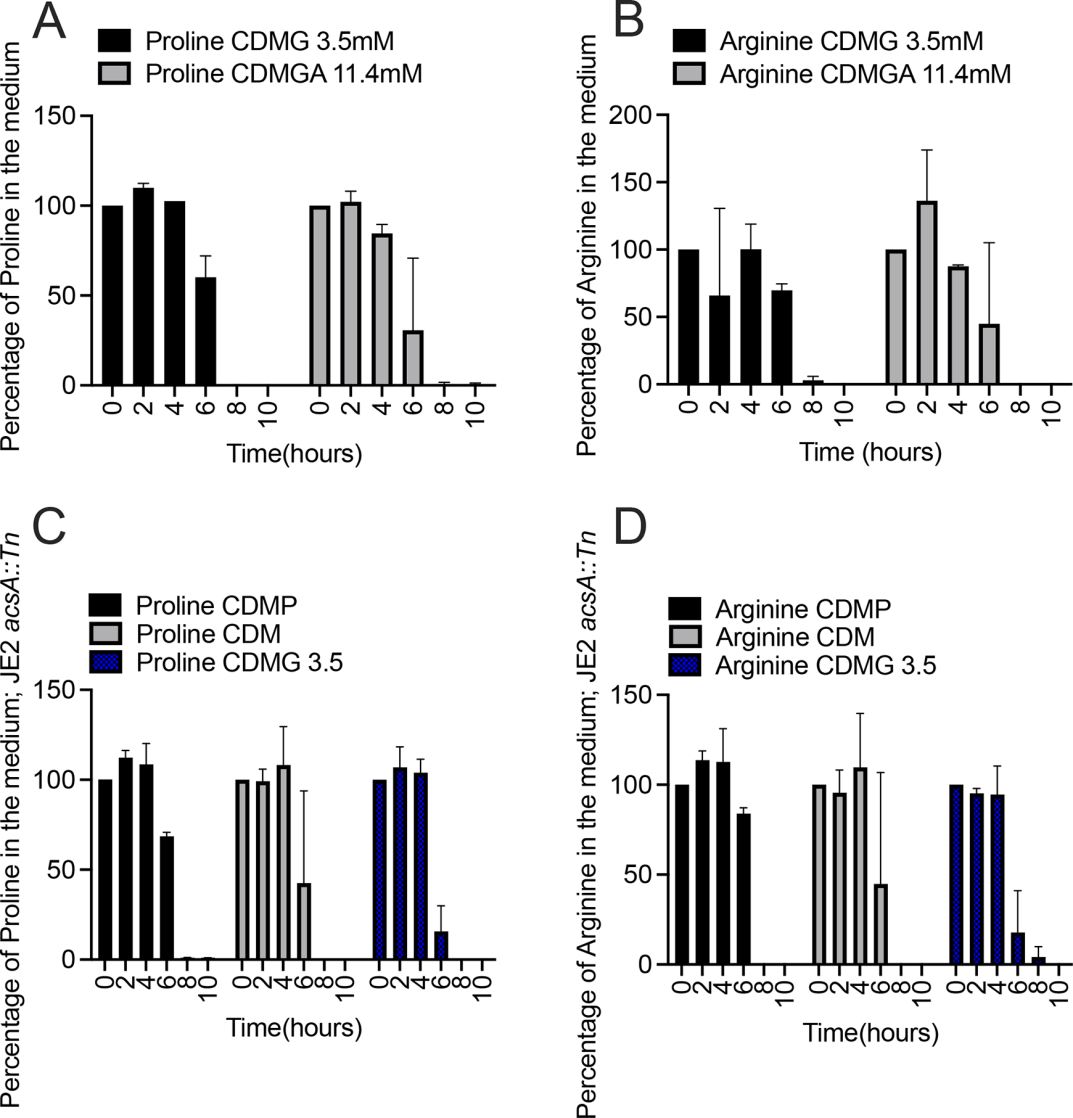
Assessment of proline and arginine in spent medium. Percentage of proline (**A**) and arginine (**B**) remaining in the medium during growth of JE2 in CDMG (3.5 mM glucose) or CDMGA (11.4 mM gluconic acid) at 0, 2, 4, 6, 8, and 10 h. Percentage of proline (**C**) and arginine (**D**) remaining in the medium during growth of JE2 *acsA::Tn* in CDMP (28 mM pyruvate), CDM, and CDMG (3.5 mM glucose).

During growth in CDM containing 14 mM glucose, we noted that not only was acetate catabolism impaired, but catabolism of proline and arginine was also inhibited, thus post-exponential growth was halted (Fig. 1D). In contrast, we found that both proline and arginine were catabolized in CDM containing 3.5 mM glucose, similar to that observed during growth in CDM, suggesting that amino acids used to fuel glutamate synthesis are consumed in this medium (Fig. 5A, B, and 1D). Indeed, the amino acid consumption profile observed in CDMG (3.5 mM) mirrored that as noted with growth in CDM except for lysine (Fig. S1 and S4).

As shown in Fig. 3E, JE2 grown in CDM with 11.4 mM gluconic acid, a non-PTS sugar, exhibited impaired acetate catabolism similar to JE2 grown in CDM with 14 mM glucose. As gluconic acid is not a PTS sugar and should not activate CcpA-mediated repression of proline and arginine catabolism, we wanted to determine if a defect in acetate catabolism would also affect catabolism of arginine and proline. As shown in Fig. 5A and B, unlike JE2 grown in CDM with 14 mM glucose, proline and arginine were completely consumed during growth in CDMGA. These results indicate that proline and arginine catabolism are independent of the ability of *S. aureus* to consume acetate from the medium. Confirming these data, both proline and arginine were catabolized when JE2 *acsA::Tn,* which is unable to consume acetate, was grown in CDM, or CDM containing 3.5 mM glucose or 28 mM pyruvate (Fig. 5C and D).

### Reduction of amino acid concentration in CDM

**Fig 6:**
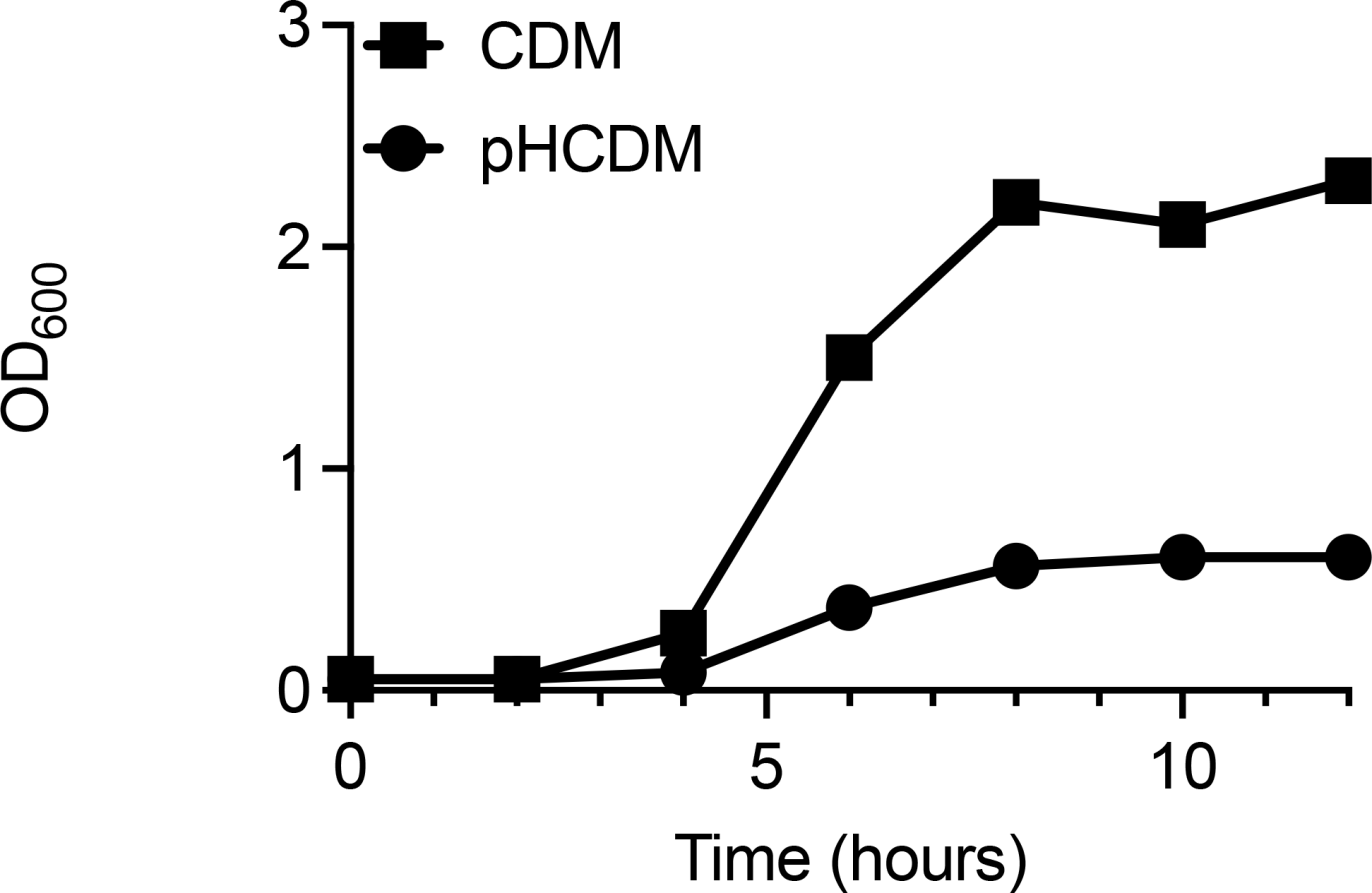
Growth of JE2 in CDM and pCDM. *S. aureus* JE2 was grown in CDM (black) or pCDM (blue) aerobically (10:1 flask to volume ratio, 250 rpm, 37°C) for 12 h.

**Fig 7:**
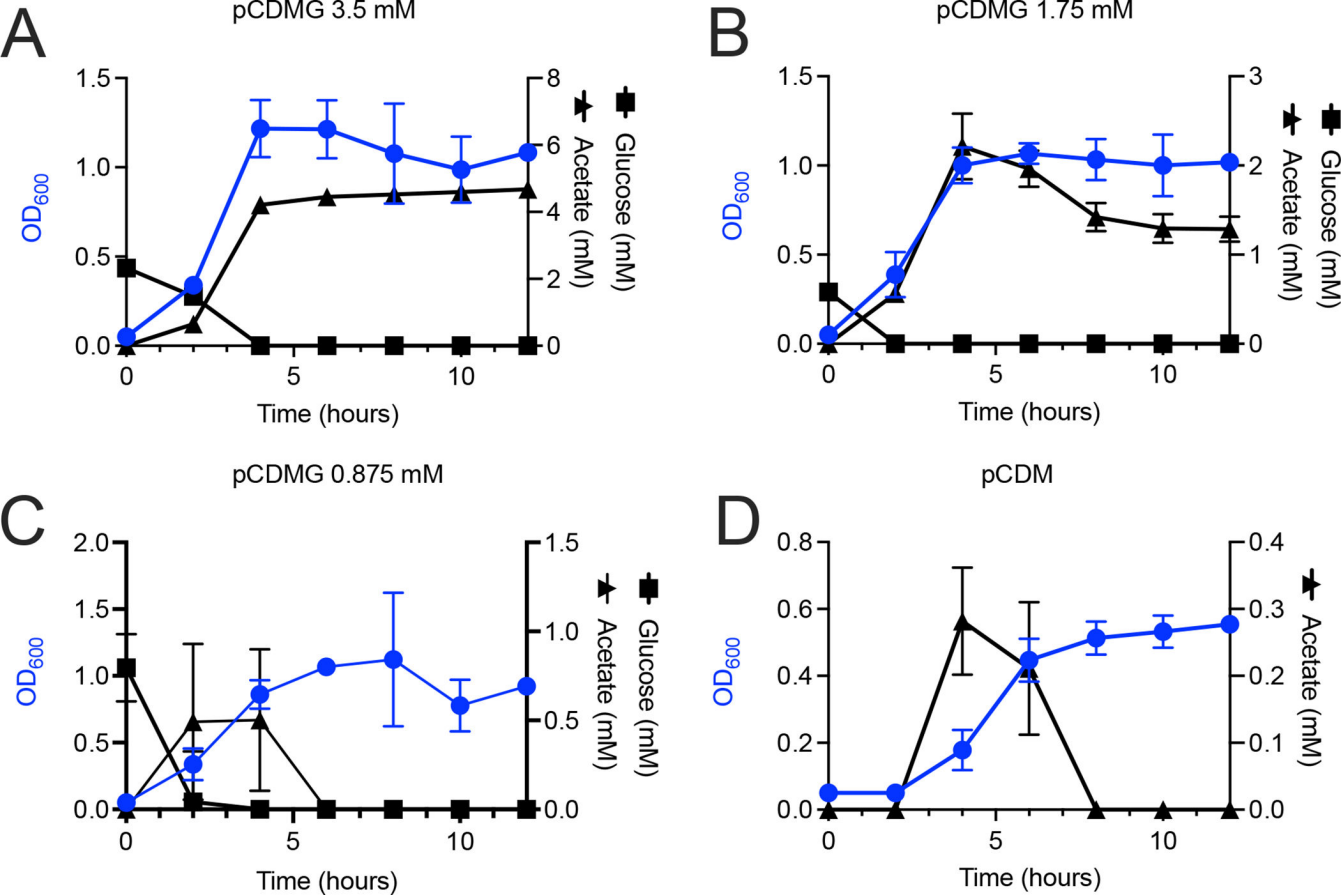
Growth of JE2 in pCDMG containing various concentrations of glucose. JE2 was grown aerobically (10:1 flask to volume ratio, 250 rpm, 37°C) for 24 h in pCDMG (3.5 mM glucose) (**A**); pCDMG (1.75 mM glucose) (**B**); pCDMG (0.875 mM glucose) (**C**); and pCDM (**D**). Extracellular acetate, glucose, and OD_600_ were assessed.

**Fig 8:**
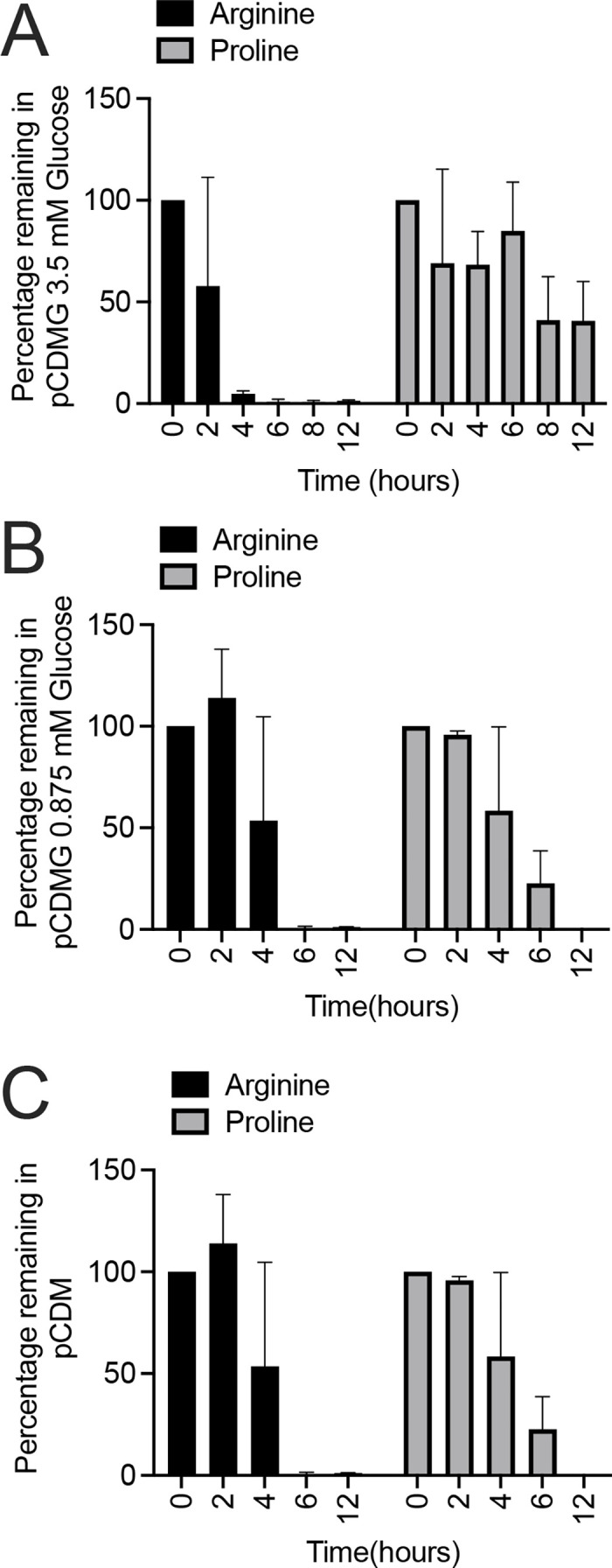
Amino acid analysis of *S. aureus* JE2 grown in pCDM containing various concentrations of glucose (pCDMG). Percentage of arginine and proline remaining in the medium during growth of JE2 in pCDMG containing 3.5 mM glucose (**A**), 0.875 mM glucose (**B**), and pCDM (**C**).

We noted that during growth in CDM or CDMG, the medium amino acid concentration as assessed at hour 2 was almost always higher than the concentration added to the original medium (Fig. 1; Fig. S1 and S2). This observation suggests that the bacterial cells were excreting amino acids into the medium, implying that the amino acid concentration in CDM is too high and potentially toxic for the cells. Furthermore, it is possible that high extracellular concentrations of amino acids may alter amino acid metabolic pathways that are utilized *in vivo,* where certain amino acids are replete and others are limited. Therefore, the amino acid concentration in CDM was reduced to mean human serum concentrations. As serum contains both glutamine and asparagine, both were added to this new medium called physiological CDM (pCDM) (Table S1). As expected, and compared to growth in CDM, the growth rate and yield of *S. aureus* in pCDM were reduced (Fig. 6). Figure S5 documents enhanced growth rate and yield when additional carbon sources, including TCA cycle intermediates, were added to pCDM. However, we were surprised to find that acetate was not catabolized in pCDM containing 3.5 mM glucose (Fig. 7A), suggesting that acetate catabolism is linked to the concentration of the glycolytic carbon source in relation to the amino acids added to the medium. Indeed, when the glucose concentration in pCDM was reduced to 0.875 mM, but not 1.75 mM (Fig. 7B), acetate catabolism was again observed (Fig. 7C). Furthermore, as predicted, acetate catabolism was observed during growth in pCDM lacking glucose (Fig. 7D). As shown in Fig. 8, amino acid analysis was performed in pCDM (3.5 mM glucose), pCDM (0.875 mM glucose), and pCDM with no glucose. Although arginine and proline were completely consumed during growth in pCDM (0.875 mM glucose) and pCDM, only arginine was completely consumed in pCDM containing 3.5 mM glucose (Fig. 8). Although unclear, this may be linked to the presence of glutamine in pCDM, which can function as a glutamate source similar to proline.

**Fig 9:**
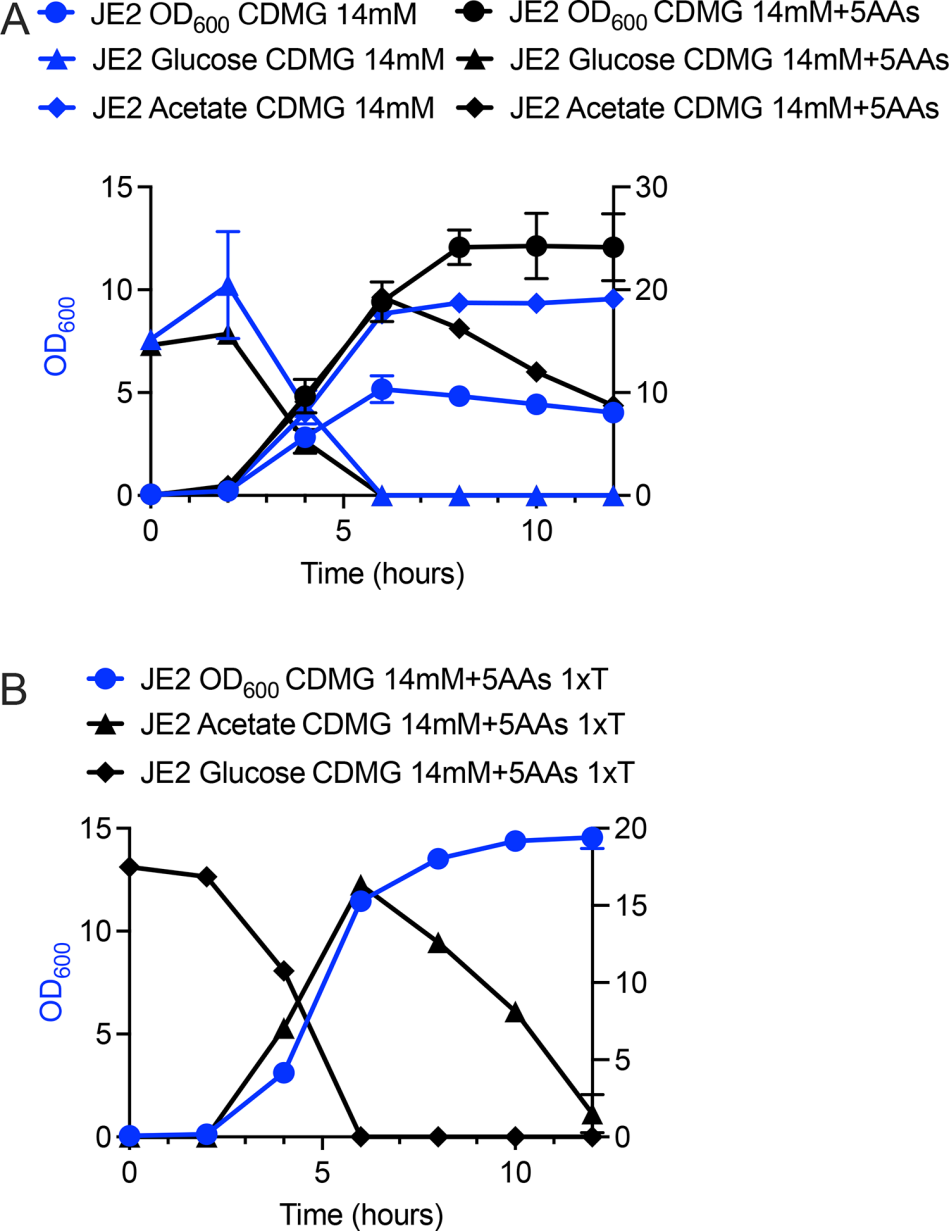
Growth of JE2 in CDMG containing 5× amino acids. JE2 was grown aerobically (10:1 flask to volume ratio, 250 rpm, 37°C) for 24 h in CDMG (14 mM glucose) with and without 5× amino acids (**A**) or 5× amino acids/1× threonine (**B**). Extracellular acetate, glucose, and OD_600_ were assessed.

Lastly, since reduction of the glycolytic source promoted acetate catabolism in medium with a reduced amino acid concentration, we hypothesized that, conversely, acetate catabolism would be observed in CDMG (14 mM glucose) if additional amino acids were added. Therefore, JE2 was grown in CDMG (14 mM) containing 5× amino acids (Fig. 9A). Although acetate was not completely consumed, extracellular acetate concentration was reduced from 20 to 10 mM. As catabolism of threonine results in extracellular acetate (15), to reduce the intracellular acetate production, JE2 was grown in CDM containing 5× amino acids/1× threonine. Interestingly, growth in CDM 5× amino acids with reduced threonine resulted in significant acetate consumption following 12 h of growth (Fig. 9B), suggesting that, as noted above, *S. aureus* acetate consumption is linked to a critical concentration of glycolytic source, amino acids, and potentially intracellular acetate concentration.

## DISCUSSION

Why do many bacterial species undergo overflow metabolism? Even in the defined media as described in this study, CDMG (3.5 mM) or pCDMG (0.875 mM), the majority of acetyl-CoA derived from pyruvate dehydrogenase during exponential growth is converted to acetate by the Pta-AckA pathway, generating ATP via substrate-level phosphorylation. It is unclear why the acetyl-CoA is not instead converted to citrate via the condensation reaction with oxaloacetate mediated by citrate synthase. The NADH produced via the TCA cycle could then drive respiration, thus generating over twice as much ATP per glucose molecule as compared to acetate fermentation. To explain these observations, several models have been proposed, primarily using *E. coli* as a model system. Basan et al. (30) and others (31) have proposed that the proteome cost of respiration exceeds that of fermentation. Others have proposed physical limitations in the membrane termed the membrane economics hypothesis (32) or the membrane real estate hypothesis (13). As bacteria grow rapidly during the exponential phase, the cells become larger, thus decreasing the surface-to-volume ratio. It is predicted that the physical space within the membrane does not allow for respiration to occur efficiently due to the number of proteins required for cytochrome activity and ATP synthesis (13). In contrast, acetate fermentation just requires glucose transport (13). Furthermore, if acetyl-CoA entered the TCA cycle during the exponential phase, the increased production of NADH may become toxic due to the lack of membrane space available for respiratory proteins to subsequently oxidize NADH to NAD^+^ (13). One of us has recently proposed a different model suggesting that acetate overflow in *S. aureus* is precipitated by a thermodynamic bottleneck in the TCA cycle, and specifically, succinate dehydrogenase (SDH) driving the succinate to fumarate reaction (14). Generation of fumarate from succinate via SDH in *S. aureus* requires the concomitant reduction of menaquinone in the respiratory chain, which has a low midpoint potential in comparison to ubiquinone. *S. aureus* does not encode ubiquinone, which is utilized to drive the SDH reaction in other organisms, such as *E. coli*.

Thus, during *S. aureus* aerobic growth, due to multiple mechanisms, including a metabolic bottleneck in the TCA cycle, acetate is excreted during the exponential phase. However, once a glycolytic or amino acid source that generates acetyl-CoA is consumed, acetate is typically consumed from the medium and enters the TCA cycle, thus generating NADH and reducing power (the “acetate switch”) (33). Data presented in this manuscript suggest that in *S. aureus,* the acetate switch is dependent upon the coordinated action of AcsA, AcuA, and AcuC. However, if the glucose concentration in the medium is too high, this process is inhibited (4, 15). We found that acetate transport and subsequent use are dependent upon a critical relationship between the concentration of amino acids and glucose in the medium. Furthermore, because catabolism of proline, arginine, and acetate in the post-exponential phase of growth is repressed by CcpA (15, 34, 35), we hypothesized that their catabolism may be linked. However, we found that although proline and arginine were catabolized when JE2 was grown in CDM containing 11.4 mM gluconic acid, which is a non-PTS sugar and thus not CcpA responsive, acetate was not consumed further, suggesting that acetate consumption is not specifically linked to CcpA and that acetate and arginine/proline catabolism are independent of one another.

Why does the addition of 28 mM pyruvate facilitate the acetate catabolism of JE2 grown in CDM, but the addition of excess gluconic acid (another non-PTS sugar) is unable? This may be linked to the decreased amount of acetate that is produced during growth in CDMP (28 mM) (~5 mM acetate; Fig. 4D) as compared to CDMG (14 mM) and CDMGA (11.4 mM) (~14 mM and ~7.5 mM acetate, respectively). These data suggest that acetate consumption is linked to the amount of acetate produced or excreted during growth; however, the concentration of acetate excreted during growth of JE2 in CDMG (3.5 mM) is similar to the amount of acetate excreted during growth in CDMGA (11.4 mM), and acetate is only consumed in CDMG (3.5 mM), suggesting that other mechanisms may be involved.

Nevertheless, during the design of growth experiments with *S. aureus,* we feel it is critically important to utilize a defined medium where the TCA cycle is active and post-exponential growth occurs using acetate, proline, and arginine as sources of glutamate. Indeed, distinct regulons are induced, such as the Agr regulon (36), during growth in exponential and post-exponential growth and in addition, the CcpA regulon is only de-repressed when glucose is consumed during post-exponential growth (37). Using acetate consumption and proline/arginine utilization as markers of post-exponential growth, our studies found use of 3.5 mM glucose was appropriate when using amino acid concentrations for CDM as defined by Hussain et al. (23).

**Fig 10:**
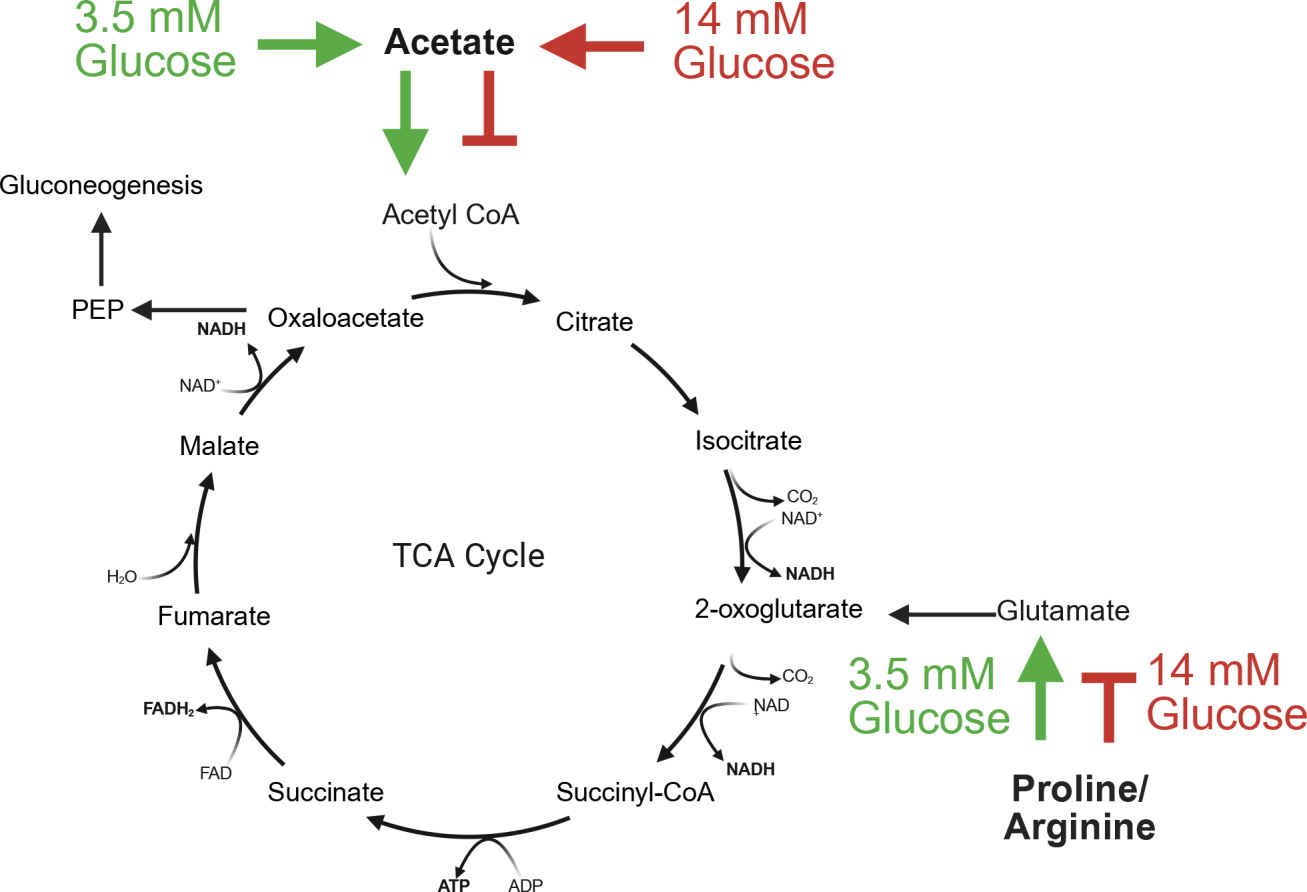
Post-exponential catabolism of acetate, proline, and arginine. Following glucose consumption and alleviation of the exponential phase, use of a high concentration of glucose (14 mM) in CDM inhibits acetate, arginine, and proline catabolism. However, the use of 3.5 mM glucose facilitates entry of acetate into the TCA cycle, generating reducing power, thus stimulating respiration. Catabolism of both proline and arginine is also observed using CDMG (3.5 mM), thus providing a critical glutamate, oxaloacetate, and gluconeogenic carbon source driving post-exponential growth.

However, the concentration of amino acids in human serum is lower than that found in the Hussain CDM recipe. Furthermore, CDM does not contain glutamine or asparagine, and glutamine is the most abundant amino acid found in serum (38). It is currently unknown if the addition of glutamine or asparagine facilitates the induction or repression of specific metabolic pathways. However, our data suggests that growth of *S. aureus* in CDM base medium results in excretion of amino acids following 2–4 h of growth. These data may suggest that the elevated concentrations of free amino acids found within base CDM are deleterious. Therefore, pCDM was developed to model more relevant (mean serum) concentrations of amino acids that also contained both glutamine and asparagine. Although the growth rate and yield are clearly different when comparing pCDM and CDM (Fig. 6 and 7), the growth yield during the exponential phase reaches an OD_600_ = 0.5, which yields enough cells to perform critical experiments, such as RNAseq (39). In conclusion, studies in this manuscript have found that acetate consumption and post-exponential growth are linked to a critical concentration between glucose and the total amino acids found in the medium. We describe that the addition of 3.5 mM glucose to CDM, as described by Hussain, or 0.875 mM glucose to pCDM, facilitates post-exponential growth as marked by acetate catabolism and proline/arginine catabolism (Fig. 10). The contribution of other metabolites and micronutrients requires further study to understand their impact on staphylococcal growth, metabolism, and physiology.

## MATERIALS AND METHODS

### Bacterial strains and growth analysis

Strains used in this study are listed in Table S1; all *S. aureus* strains are derived from *Staphylococcus aureus* USA300 JE2. *S. aureus* strains were either grown aerobically (10:1 flask to volume ratio, 250 rpm, 37°C) or in flat, transparent 96-well microtiter plates (Thermofisher) using Tecan Infinite M200 spectrophotometer (program settings: plate with cover, 37°C, kinetic cycle: 40–48, kinetic interval: 30 min, absorbance—600 nm/number of flashes: 3–4, shaking 1—time [s]—900 and shaking 2—time [s]—860). Overnight cultures for use in Tecan growth experiments were grown for 12–14 h in 3 mL TSB, washed once in 0.85% saline, and used to inoculate the media at a starting optical density at 600 nm (OD_600_) of 0.05. JE2 and corresponding mutants were grown in CDM (15, 23) supplemented with D-glucose (14 or 3.5 mM), sodium pyruvate (28 mM), or D-gluconic acid sodium salt (2.8 or 11 mM).

Plasmids were isolated using the Zymo Research Plasmid Miniprep Kit (Orange, CA). Gel elution of PCR fragments was performed using Wizard SV gel and PCR clean-up system from Promega (Madison, WI). Genomic DNA isolation from *S*. *aureus* strains was performed using the Wizard genomic DNA purification kit from Promega. Antibiotics were purchased from Sigma Aldrich (St. Louis, MO), and concentrations used were carbenicillin 100 ug/mL, erythromycin 10 ug/mL, kanamycin (75–100 ug/mL), spectinomycin 1,000 ug/mL, tetracycline 10 ug/mL, chloramphenicol 10 ug/mL, and trimethoprim 10 ug/mL.

### Genetic manipulation of *S. aureus*

Bacteriophage Φ11 was used to transduce appropriate markers into JE2 or other strains of interest. Transposon insertions were verified using PCR amplification of target loci. To generate JE2 *acuC::Tn acuA::dhfr,* the erythromycin resistance cassette in the JE2 *acuA*::Tn mutant was exchanged to a trimethoprim resistance cassette, generating JE2 *acuA::tmp* as described (40). Briefly, the plasmid psDhfr-FR (8.8 kb) was generated as follows: a cassette containing P*sarA* driving a gene encoding trimethoprim resistance (*dhfr*) was PCR amplified with primers FR 12 and FR 13, and genomic DNA isolated from a *pyrE* mutant strain (P. D. Fey, unpublished data) was used as a template. Midas Mix (Monserate Biotechnology Group, CA) was used for PCR amplifications as per the manufacturer’s method. The PCR fragment with a size of 789 bp was cloned into pCR2.1 Topo vector (Invitrogen), and transformation was performed using chemically competent *E. coli* DH5α (Invitrogen). The recombinant plasmid was sequenced via Eurofins Genomics (Louisville, KY) using vector-specific M13 forward and reverse primers. The P*sarA dhfr* (s*dhfr*) fragment in the Topo vector was digested with XbaI-HF restriction enzyme and ligated with NheI-HF digested- and shrimp alkaline phosphatase- (rSAP[1,000 U/mL], Biolab; 1 μL added in enzyme mix incubated at 37°C/1 h) treated pTnT vector (40). All the restriction enzymes and ligases were purchased from New England Biolabs, Beverly, MA. The cloning of the *dhfr* antibiotic resistance cassette (encoding trimethoprim resistance) into the pTnT vector (temperature sensitive) was performed using primers FR14 and FR15. The resulting plasmid was named pFR5, and the orientation of the cassette was confirmed using primers FR 16 and JBTN17 (40). The pFR5 plasmid was electroporated into *S. aureus* RN4220 and plated on TSA containing 10 mg/μL of chloramphenicol (incubated at 30°C). Subsequent transduction of the resulting plasmid was performed via f11-mediated transduction. This was followed by an allelic exchange procedure previously described (40). The resultant JE2 *acuA::*Tmp strains were screened for trimethoprim resistance (Tmp) and were susceptible to erythromycin and chloramphenicol. Further confirmation of antibiotic marker exchange was done by sequencing the PCR amplified product with a size of 1.6 kb using primers 3413/FR12. The JE2 *acuA::*Tmp mutation was transduced into JE2 *acuC::*Tn mutant by bacteriophage Φ11-mediated transduction.

Chromosomal complementation of JE2 *acsA::Tn* and JE2 *acuC::Tn* mutants was done following the protocol for single-copy vectors for integration at the SaPI1 attachment site for *S. aureus* (41). The *sarA* promoter is used to express the *acuC* gene. The fragment with the *sarA* gene was synthetically made from IDT. Fragment (s*acuC*FR) and shuttle vector pJC1111 digested with PstI and BamHI enzymes were assembled using Gibson assembly (Q5 high fidelity 2× Master mix-NEB). The Gibson reaction mix of 1:10 dilution was electroporated into E10B (*E. coli*) cells. The transformation mix was plated onto LB amp (50 ug/mL). The recombinant vector pFR7 carrying *E. coli* cells was processed for plasmid isolation and sequenced with oLH 229-230 primers for confirmation. After sequence confirmation, the pFR7 vector was electroporated into RN9011 with the pRN7023 plasmid integrase for chromosomal integration. Cells were plated onto TSA with 0.1 mM cadmium chloride. The integration of the complementation cassette at the SaPI1 site was confirmed with PCR using JCO 717 and 719 primers, yielding a 1.1 kb amplification product (41). Phage 11-mediated transduction was used to move the integrated complementation cassette into the JE2 *acuC::*Tn mutant strain to generate the JE2 *acuC* pFR7::SapI1 strain. The control for this complementation, the empty pJC1111, was integrated chromosomally and named as JE2 *acuC* pJC1111::SapI1.

The complemented strain for JE2 *acsA*::Tn was generated in a similar way, except that the native *acsA* promoter was used. The fragment n*acsA*FR was amplified using FR21 and FR22 primers, and JE2 genomic DNA was used as a template. Cloning of this fragment into pJC1111 was similar to that noted above. The complemented strain generated was JE2 *acsA* pFR8::SaPI1, and the control strain JE2 *acsA* pJC1111::SaPI1.

### Acetate, glucose, and amino acid analysis

Extracellular acetate/glucose in spent medium was measured by collecting 1 mL of medium, followed by centrifugation for 10 min at 14,000 rpm at room temperature. The supernatants were stored at −80°C until analyzed using kits purchased from R-Biopharm (Pfungstadt, Germany) per the manufacturer’s protocol.

Concentration of amino acids in spent medium was assessed by first growing strains to test overnight in 3 mL TSB at 37°C with shaking at 250 rpm for 14–16 h. The cell pellet was washed with 0.85% saline and inoculated at a starting cell density of *A*_600_ = 0.05 into CDM and subsequently grown aerobically (250 rpm; 10:1 flask to volume ratio, 37°C). One milliliter of spent culture medium was collected every 2 h for 14 h and centrifuged for 5 min at 14,000 rpm at room temperature. Supernatant was subsequently diluted 1/100 in diluent (10 mM ammonium acetate, 10 mM ammonium hydroxide [NH_4_OH], and 5% acetonitrile). Blank used in the analysis was CDM, which was diluted 1/50 in the same diluent. All the diluted samples (500 uL) were filtered using Amicon Ultra centrifugal filters (Millipore-3,000 molecular weight cut off). The final volume of filtered samples used for liquid chromatography-tandem mass spectrometry (LC-MS/MS) was 200 uL. LC-MS/MS separation and quantitation were carried out using an XBridge Amide 3.5 µm (2.1 × 100 mm) column procured from Waters. Mobile phase A was 10 mM ammonium acetate and 10 mM ammonium hydroxide, 5% acetonitrile in water, whereas mobile phase B was 100% acetonitrile. The flow rate was 0.4 mL/min with a gradient mode of mobile phases. The gradient was started with the A/B solvent ratio at 10/90, followed by a gradual increase of A to 30% for 2.5 min, A was increased to 45% for 1.5 min, then to 75% 1.5 min and finally to 95% over 1.5 min. A was maintained at 95% over 6 min and then reduced to 10% A within 0.5 min and was equilibrated for 8.0  min before the next run. The column was maintained at 40°C. Detection and quantification of metabolites was carried out using QTRAP 6500 plus (Sciex, USA) in multiple reaction mode. A calibration curve was constructed using amino acid standards. Cultures were grown in triplicate in every medium and proceeded for intracellular metabolite assay as described previously (42).

### Quantitative PCR analysis

Bacterial cells were aerobically grown in 250 mL flasks, and cells were harvested (4 mL) during the post-exponential phase (OD ~1.0 nm). Cell culture was treated with an equal volume of RNA protect solution (Qiagen), and the culture was centrifuged at 5,000 rpm/4°C/10 min, and the cell pellet was stored at −80°C for later use. RNA extraction was performed using the RNeasy Mini kit (Qiagen) using the manufacturer’s protocol, including on-column DNase treatment (RNAse-Free DNase kit-Qiagen) according to the manufacturer’s instructions. RNA integrity was examined visually by agarose gel electrophoresis, and RNA concentration was determined using Nanodrop. Using Superscript IV VILO Master mix with ezDNase, cDNA was synthesized. cDNA was diluted 1:10, and 4 uL diluted cDNA was used with 10 uM primers and 5 uL SYBR mix in a total volume of 10 uL. Reactions were run using a MicroAmp optical 96-well plate with adhesive film PCR/real-time compatible (Applied Biosystems). Experiments were performed using the QuantStudio 3 machine (Applied Biosystems). The parameters used were 95 (30 s), 60 (30 s), 72 (1 min) total of 40 cycles. The constitutively expressed *gyrB* gene was used as an internal control; primers used for *gyrB* were OLH 17/18, and target genes were amplified using *acsA*-qFR1/2, *acuA*-qFR3/4, and *acuC*-qFR5/6 primers.

### Statistical analysis

All graphs were generated using GraphPad Prism 5.0 software. Metabolite one-factor statistical analysis was performed using the normalized mean peak intensities for each metabolite identified from a triplicate set of LC-MS/MS experiments. Metabolite fold change, VIP score, and subsequent *t*-test were performed using https://www.metaboanalyst.ca/.

## SUPPLEMENTAL MATERIAL

Fig. S1 aem.01554-25-s0001.tif Amino acid analysis of *S. aureus* JE2 grown in CDM.

Fig. S2 aem.01554-25-s0002.tif Amino acid analysis of *S. aureus* JE2 grown in CDM containing 14 mM glucose (CDMG).

Fig. S3 aem.01554-25-s0003.tif Transcriptional analysis of *acsA*, *acuA*, and *acuC* in JE2 *ccpA::tetL*.

Fig. S4 aem.01554-25-s0004.tif Amino acid analysis of *S. aureus* JE2 grown in CDM containing 3.5 mM glucose (CDMG).

Fig. S5 aem.01554-25-s0005.tif Addition of various carbon sources to pCDM and assessment of growth.

Table S1 aem.01554-25-s0006.docx CDM(G) and pCDM(G) recipe.

Table S2 aem.01554-25-s0007.docx Strains, plasmids, and primers used in this study.

ASM does not own the copyrights to Supplemental Material that may be linked to, or accessed through, an article. The authors have granted ASM a non-exclusive, world-wide license to publish the Supplemental Material files. Please contact the corresponding author directly for reuse.
