# Supplementary figures and images for: Growth and acetate metabolism of Staphylococcus aureus in defined medium
Source: Appl Environ Microbiol. 2025 Oct 16;91(11):e01554-25. doi: 10.1128/aem.01554-25 (PMC12628684; doi:10.1128/aem.01554-25)

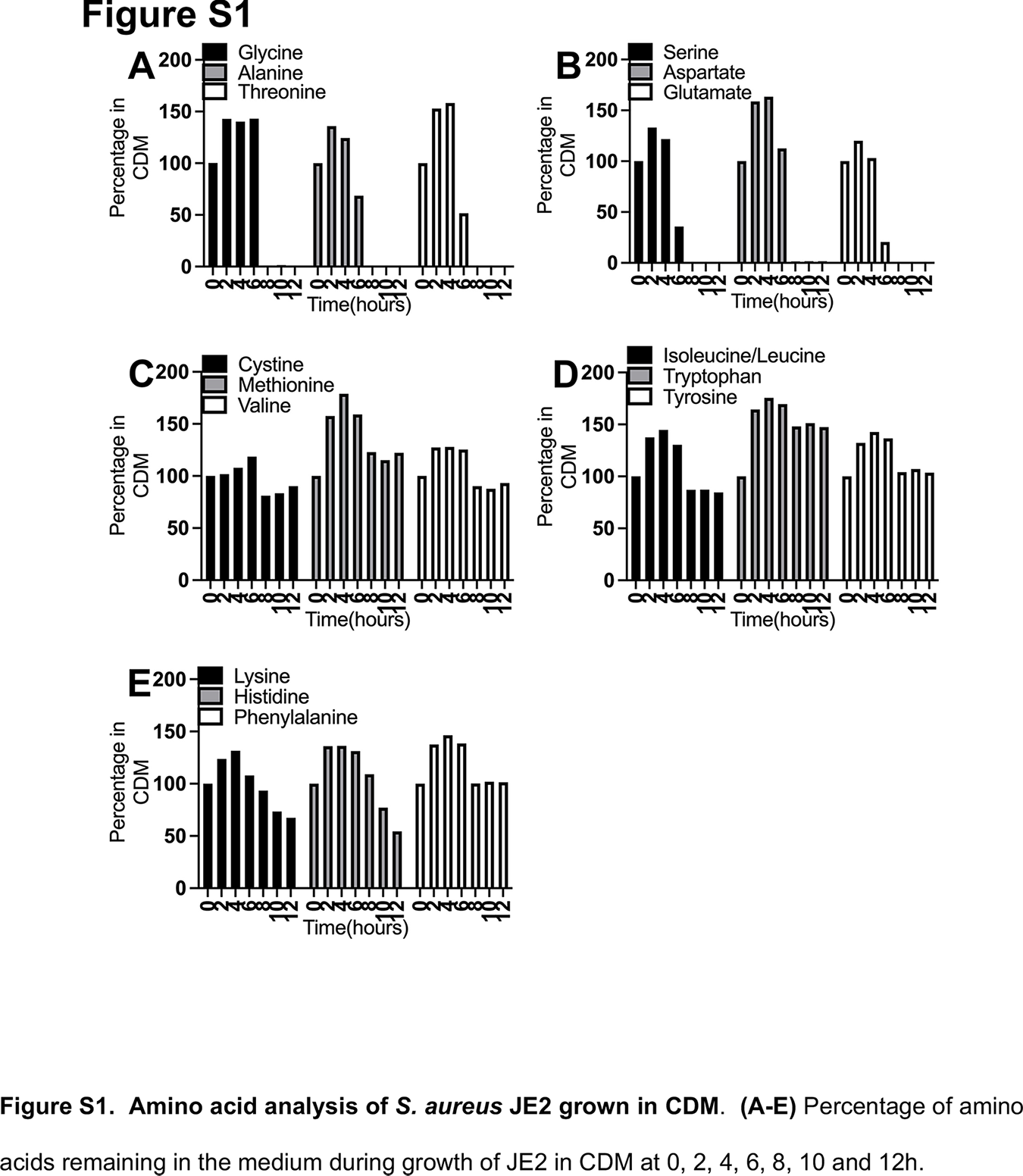

Supplement: Fig. S1 — Amino acid analysis of S. aureus JE2 grown in CDM. [file aem.01554-25-s0001.tif]

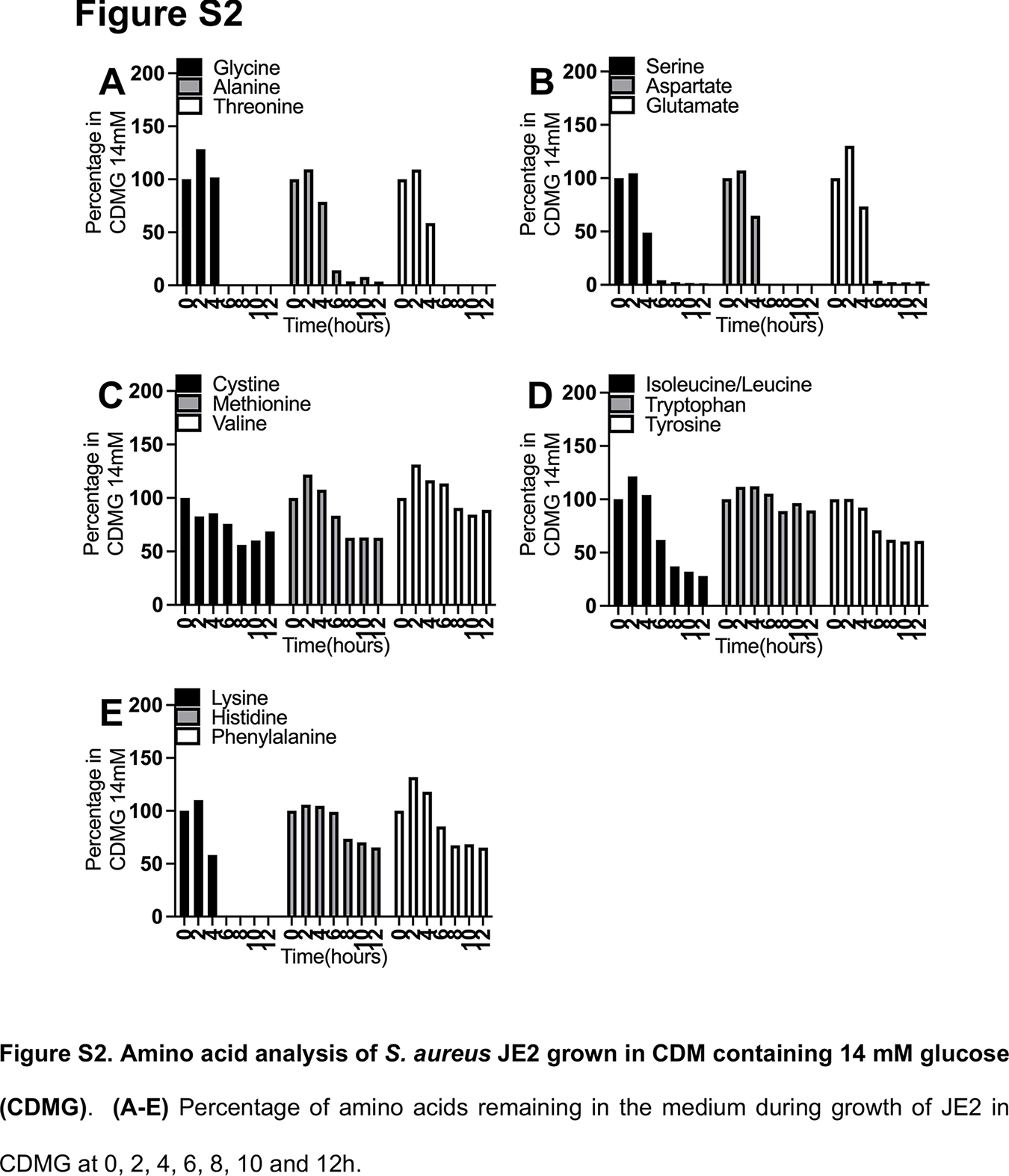

Supplement: Fig. S2 — Amino acid analysis of S. aureus JE2 grown in CDM containing 14 mM glucose (CDMG). [file aem.01554-25-s0002.tif]

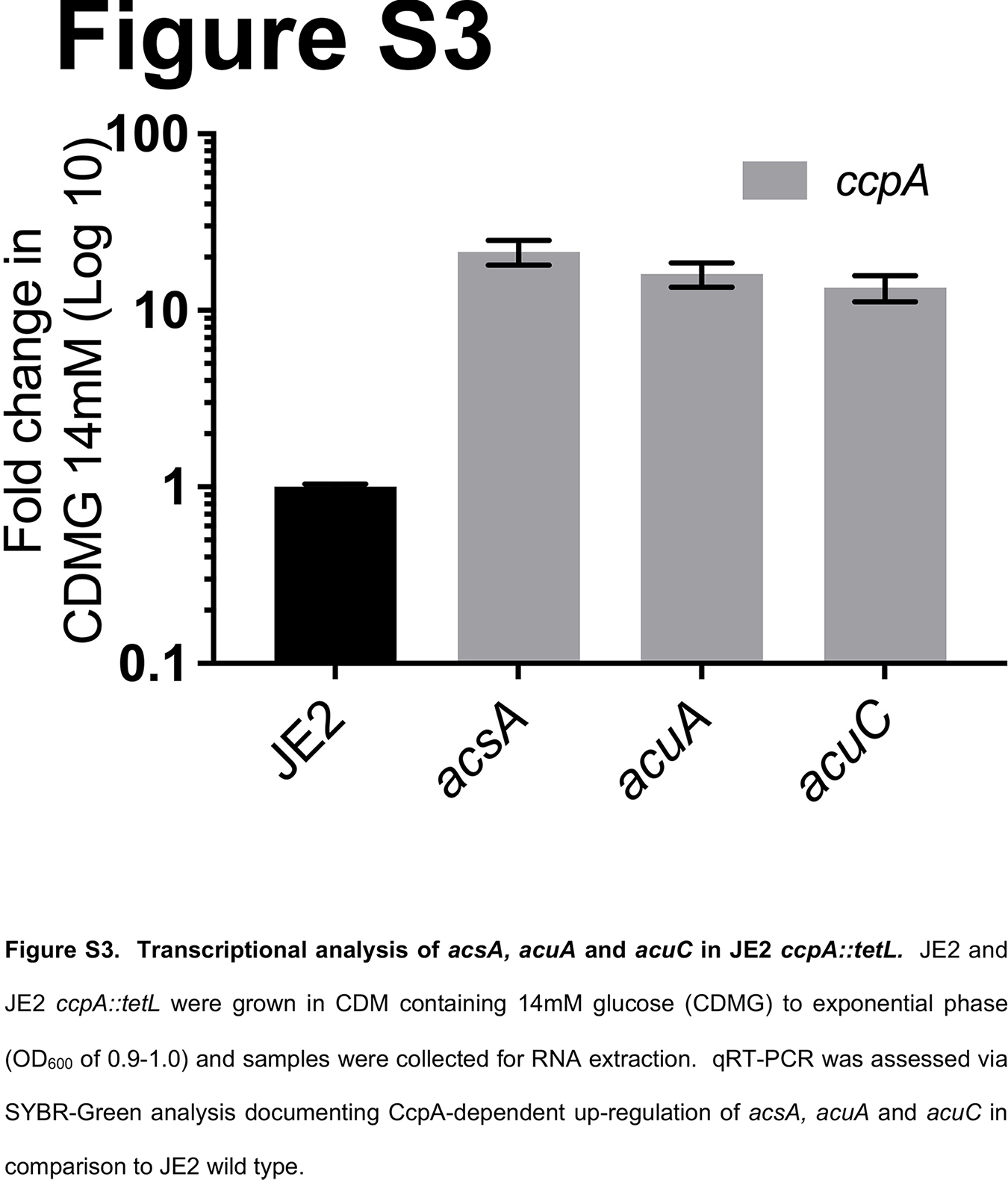

Supplement: Fig. S3 — Transcriptional analysis of acsA, acuA, and acuC in JE2 ccpA::tetL. [file aem.01554-25-s0003.tif]

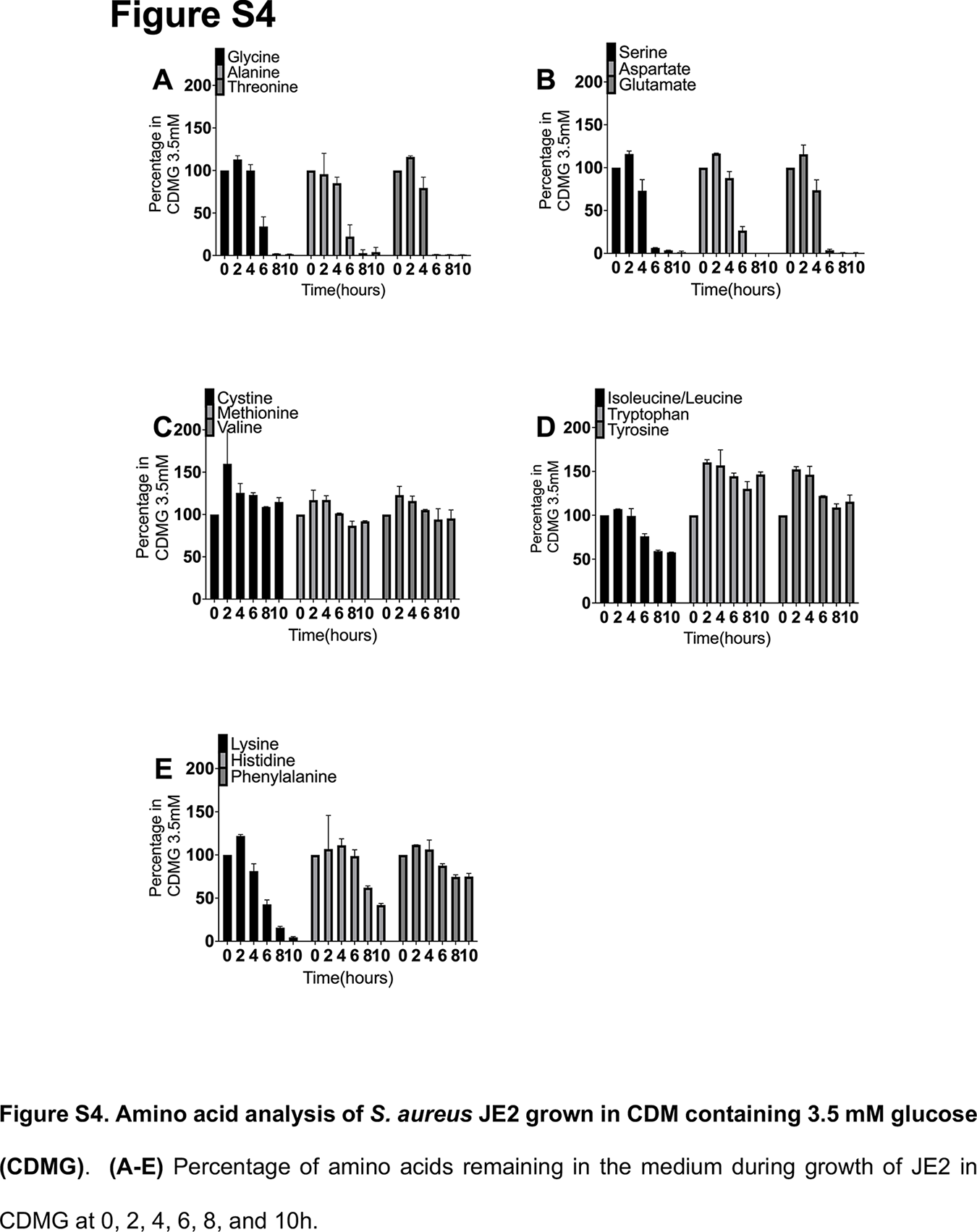

Supplement: Fig. S4 — Amino acid analysis of S. aureus JE2 grown in CDM containing 3.5 mM glucose (CDMG). [file aem.01554-25-s0004.tif]

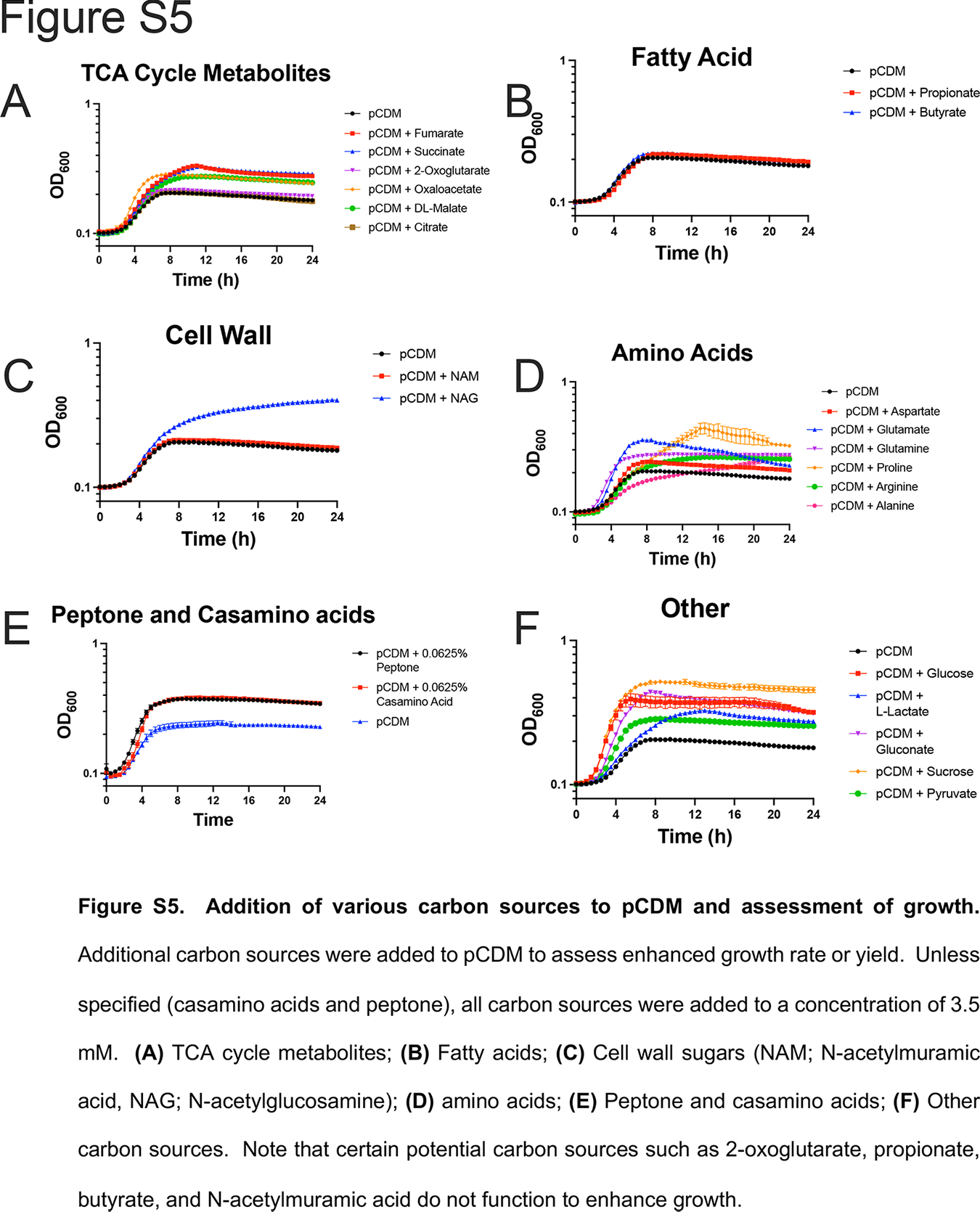

Supplement: Fig. S5 — Addition of various carbon sources to pCDM and assessment of growth. [file aem.01554-25-s0005.tif]
