# Supplementary material for: Growth and acetate metabolism of Staphylococcus aureus in defined medium
Source: Appl Environ Microbiol. 2025 Oct 16;91(11):e01554-25. doi: 10.1128/aem.01554-25 (PMC12628684; doi:10.1128/aem.01554-25)
Supplement: Table S1 — CDM(G) and pCDM(G) recipe. [file aem.01554-25-s0006.docx]

**Table S1**

**CDM(G) and pCDM(G) recipe**

**GROUP 1**

Amino acid CDM mg (μmolar) pCDM mg (μmolar)

L-alanine 100 (1122) 45.4 (505)

L-arginine 100 (574) 11.1 (64)

L-aspartate 150 (1127) 0.80 (6)

L-cystine 50 (208) 8.7 (36)

L-glutamate 150 (1020) 14.3 (98)

glycine 100 (1332) 24.8 (330)

L-histidine 100 (644) 18.6 (120)

L-isoleucine 150 (1144) 13.1 (100)

L-leucine 150 (1144) 22.3 (170)

L-lysine 100 (547) 32.2 (176)

L-methionine 100 (670) 4.4 (30)

L-phenylalanine 100 (605) 11.2 (68)

L-proline 150 (1303) 41.4 (360)

L-serine 100 (952) 14.7 (140)

L-threonine 150 (1259) 28.6 (240)

L-tryptophan 100 (490) 17.0 (83)

L-tyrosine 100 (552) 13.4 (74)

L-valine 150 (1280) 36.3 (310)

L-glutamine Not added 116.8 (800)

L-asparagine Not added 12.0 (90)

Na_2_HPO_4_ 10000 mg 10000 mg

KH_2_PO_4_ 3000 mg 3000 mg

Add Group 1 components to 700 ml water. Use a hot plate (~80°C) and slowly stir to dissolve the solution and filter sterilize. Make Fresh to use same day.

**GROUP 2**

Glucose (if required) 625 mg (3.5 mM) 156 mg (0.875 mM)

MgSO_4_•7H_2_O 500 mg 500 mg

Add Group 2 components to 100 ml water and filter sterilize. Make Fresh to use same day.

**GROUP 3 (100x)**

Biotin 10 mg 10 mg

Nicotinic acid 200 mg 200 mg

D-Pantothenic acid, 200 mg 200 mg

hemi Ca Salt

Pyridoxal hydrochloride 400 mg 400 mg

Pyridoxamine 400 mg 400 mg

dihydrochloride

Riboflavin 200 mg 200 mg

Thiamin hydrochloride 200 mg 200 mg

Add Group 3 components to 1000 ml water and filter sterilize. Keep refrigerated, light sensitive.

**GROUP 4 (20x)**

Adenine sulphate 400 mg 400 mg

Guanine hydrochloride 400 mg 400 mg

Add Group 4 components to 1000 ml water, stir and add NaOH pellets to dissolve the salts. Filter sterilize and keep refrigerated.

**GROUP 5 (100x)**

CaCl_2_•6H_2_O 1000 mg 1000 mg

MnSO_4_ 500 mg 500 mg

FeSO_4_•7H_2_O 600 mg 600 mg

Add Group 5 components to 1000 ml water and filter sterilize. Keep refrigerated, light sensitive.

**RECIPE**

Into 1 L sterile flask containing filter sterilized group 1 (700 ml)

Add:

100 ml filter sterilized Group 2

10 ml filter sterilized Group 3

50 ml filter sterilized Group 4

10 ml filter sterilized Group 5

Q.s. to 1L with sterile water (~130 ml); pH 7.2
