# Supplementary material for: Growth and acetate metabolism of Staphylococcus aureus in defined medium
Source: Appl Environ Microbiol. 2025 Oct 16;91(11):e01554-25. doi: 10.1128/aem.01554-25 (PMC12628684; doi:10.1128/aem.01554-25)
Supplement: Table S2 — Strains, plasmids, and primers used in this study. [file aem.01554-25-s0007.docx]

**Table S2. Strains, plasmids and primers used in study**

**Strain Relevant Feature Reference**

*S. aureus* JE2 *S. aureus* LAC cured of 3 plasmids; Erm^S^; Cm^S^; Tet^S^ (1)

*S. aureus* RN4220 Restriction deficient NCTC 8325-4 (2)

*S. aureus acsA::Tn bursa aurealis* Tn mutant in *acsA* (SAUSA300_1679) (1); This study

*S. aureus acuC::Tn bursa aurealis* Tn mutant in *acuC* (SAUSA300_1681) (1); This study

*S. aureus acuA::Tn bursa aurealis* Tn mutant in *acuA* (SAUSA300_1680) (1); This study

*S. aureus gltA::Tn bursa aurealis* Tn mutant in *gltA* (SAUSA300_1641) (1); This study

*S. aureus codY::Tn bursa aurealis* Tn mutant in *codY* (SAUSA300_1148) (1); This study

*S. aureus sirTM::Tn bursa aurealis* Tn mutant in *sirTM* (SAUSA300_0327) (1); This study

*S. aureus cobB::Tn bursa aurealis* Tn mutant in *cobB* (SAUSA300_2157) (1); This study

*S. aureus ccpA::tet ccpA* allelic replacement mutant; Tet^R^ (3)

*S. aureus acuA::tmp bursa aurealis* Tn mutant in *acuA.* Erm cassette exchanged (4); This study

with *dhfr;* Tmp^R^

*S. aureus* RN9011 RN4220 containing pRN7023 encoding SaPI1 integrase; Cm^R^ (5)

*S. aureus* JE2 *acuC*/ pJCs*acuC* integrated into the SaPI1 integration site This study

pJCs*acuC::sapI1*

*S. aureus* JE2 *acuC*/ pJC1111 integrated into the SaPI1 integration site This study

pJC1111*::sapI1*

*S. aureus* JE2 *acsA*/ pJCn*acsA* integrated into the SaPI1 integration site This study

pJCn*acsA::sapI1*

*S. aureus* JE2 *acsA*/ pJC1111 integrated into the SaPI1 integration site This study

pJC1111::*sapI1*

*E. coli* DH10B Cloning host Stratagene

*E. coli* DH5α Cloning host Gibco; BRL

**Plasmid Relevant Feature Reference**

pSDhfr pTnT with *dhfr* driven by the sarA promoter cloned at the Nhe1 (4); This study

site

pJC1111 SaPI1 *attS* suicide vector*,* cadmium resistant (*cadCA*) (6)

pJCs*acuC acuC* driven by the *sarA* promoter. Synthetic fragment assembled This study

via Gibson assembly into the Pst1-BamH1 site of pJC1111

pJCn*acsA acsA* driven by its native promoter. Synthetic fragment assembled This study

via Gibson assembly into the Pst1-BamH1 site of pJC1111

**Primer Primer sequence (5’🡪3’) Reference**

3108 CGAAGTTTATAAAGGAGCGCAAGG This study

3109 GCCATGTTCATTTTTCCAGTTTCAC This study

3413 GCGCATTTAACCGCATTTAG This study

3414 TGTTGTTGCATATAATCCCTCC This study

3415 TCGATTTCATGACCAACATCC  This study

3416 CCACCACCTAAGTGACAACC This study

M13 TGTAAAACGACGGCCAGT This study

M14 CAGGAAACAGCTATGACC This study

FR 12 GCTGCCTCTAGAGCTGATATTTTTGACTAAACCA This study

FR 13 GCTGCTGTCTAGAATTCGGCTCGAGCTATT This study

FR 14 GGACACAAATTGGAATACAAC This study

FR 15 GACGATATTCTCGATTGACCC This study

F-FR 21 AGCTGGCGGCCGCTGCATGCCTGCAGATTTTCAACT This study

CCTCAATCG

R-FR 22 CCTGAATTCGAGCTCGGTACCCGGGGATCCTTATTCC This study

ATTGTACTTAAATCCC

OLH 229 CTCACATGTTCTTTCCTGCG This study

OLH230 CAAAATTATACATGTCAACG This study

JCO 717 (3417) GTGCTTCACCAGCACCACATGCTG (5)

JCO 719 (3418) GGTATTAGTTTGAGCTGTCTTGGTTCATTGATTGC (5)

1. Fey PD, Endres JL, Yajjala VK, Widhelm TJ, Boissy RJ, Bose JL, Bayles KW. 2013. A genetic resource for rapid and comprehensive phenotype screening of nonessential *Staphylococcus aureus* genes. mBio 4:e00537-12.

2. Kreiswirth BN, Lofdahl S, Betley MJ, O'Reilly M, Schlievert PM, Bergdoll MS, Novick RP. 1983. The toxic shock syndrome exotoxin structural gene is not detectably transmitted by a prophage. Nature 305:709-12.

3. Nuxoll A, Halouska S, Sadykov M, Hanke M, Bayles KW, Kielian T, Powers R, Fey PD. 2012. CcpA regulates Arginine Biosynthesis in *Staphylococcus aureus* through repression of proline catabolism. PLoS Pathogens 8:e1003033.

4. Bose JL, Fey PD, Bayles KW. 2013. Genetic Tools To Enhance the Study of Gene Function and Regulation in *Staphylococcus aureus*. Appl Environ Microbiol 79:2218-24.

5. Chen J, Yoong P, Ram G, Torres VJ, Novick RP. 2014. Single-copy vectors for integration at the SaPI1 attachment site for *Staphylococcus aureus*. Plasmid 76:1-7.

6. Geisinger E, George EA, Chen J, Muir TW, Novick RP. 2008. Identification of ligand specificity determinants in AgrC, the *Staphylococcus aureus* quorum-sensing receptor. J Biol Chem 283:8930-8.
